# Supplementary material for: Deficiency in BDNF/TrkB Neurotrophic Activity Stimulates δ-Secretase by Upregulating C/EBPβ in Alzheimer’s Disease
Source: Cell Rep. Author manuscript; Available in PMC 2019 Aug 6. (PMC6684282; doi:10.1016/j.celrep.2019.06.054)
Supplement: 1 [file NIHMS1535616-supplement-1.pdf]

Cell Reports, Volume *28*

Supplemental Information

Deficiency in BDNF/TrkB Neurotrophic

Activity Stimulates δ-Secretase

by Upregulating C/EBPβ in Alzheimer's Disease

Zhi-Hao Wang, Jie Xiang, Xia Liu, Shan Ping Yu, Fredric P. Manfredsson, Ivette M. Sandoval, Shengxi Wu, Jian-Zhi Wang, and Keqiang Ye

Supplementary Figure 1

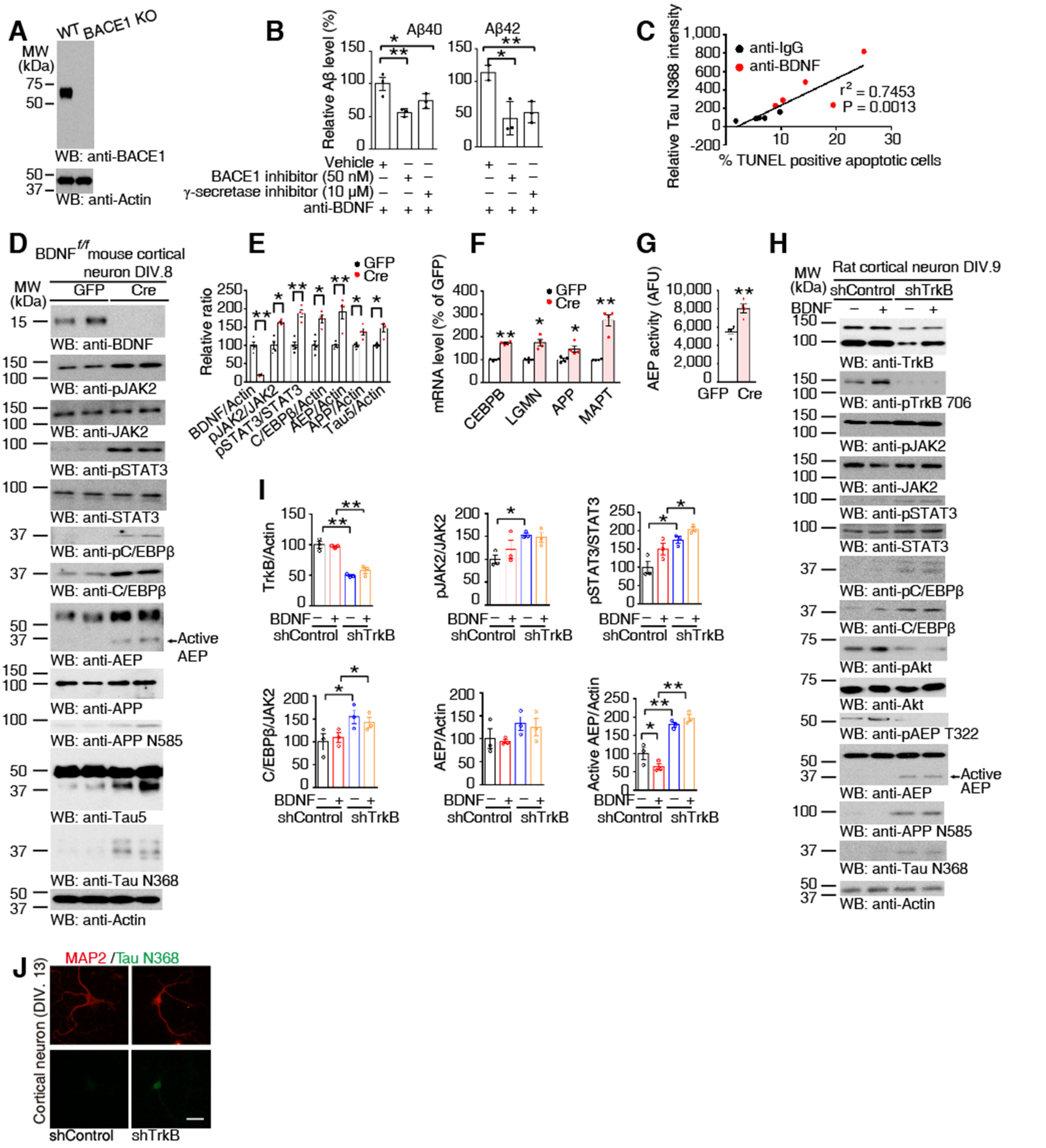

Fig. S1 BDNF/TrkB depletion activates JAK2/STAT3, increasing C/EBP expression. Related to Figure 1.

A-B Validation of anti-BACE1 antibody and Aβ ELISA kits (mean ± s.e.m., n = 3, *P < 0.05, **P < 0.01, one-way ANOVA and Bonferroni's multiple comparison test and Bonferroni's multiple comparison test). **C** TUNEL and immunofluorescence double-labeling assay showing BDNF deprivation-induced neurotoxicity

directly correlates with Tau N368 level. The Spearman correlation coefficient r^2 and *p* value are shown. **D** BDNF depletion activates JAK2/STAT3 and increases the expressions of C/EBPβ and δ-secretase. Immunoblotting was conducted from $BDNF^{f/f}$ mice neurons transduced with AAV-Cre or AAV-GFP. Western blot data are representative of three independent experiments. **E** Quantification of western blotting results in Fig. S1D (mean ± s.e.m., n = 4, *P < 0.05, **P < 0.01, unpaired t-test with Welch's correction). **F** Quantitative RT-PCR analysis of CEBPB, LGMN, APP and MAPT mRNA levels in $BDNF^{f/f}$ neurons transduced with AAV-Cre or AAV-GFP. Data represent mean ± s.e.m. (n = 4, *P < 0.05, **P < 0.01, unpaired t-test with Welch's correction). **G** BDNF deprivation stimulates δ-secretase enzymatic activities. Data represent mean ± s.e.m. (n = 4, **P < 0.01, unpaired t-test with Welch's correction). **H** TrkB depletion activates JAK2/STAT3 and increases the expression of C/EBPβ and δ-secretase. Immunoblotting was conducted from neurons transduced with vectors expressing shRNA against TrkB or control shRNAs. BDNF (100 ng/ml) was supplied for 20 minutes before cell harvest. Western blot data are representative of three independent experiments. **I** Quantification of western blotting results (mean ± s.e.m., n = 3, **P < 0.01, unpaired t-test with Welch's correction). **J** IF staining showing an increase of Tau cleavage induced by TrkB depletion. Scale bar, 30 µm.

Supplementary Figure 2

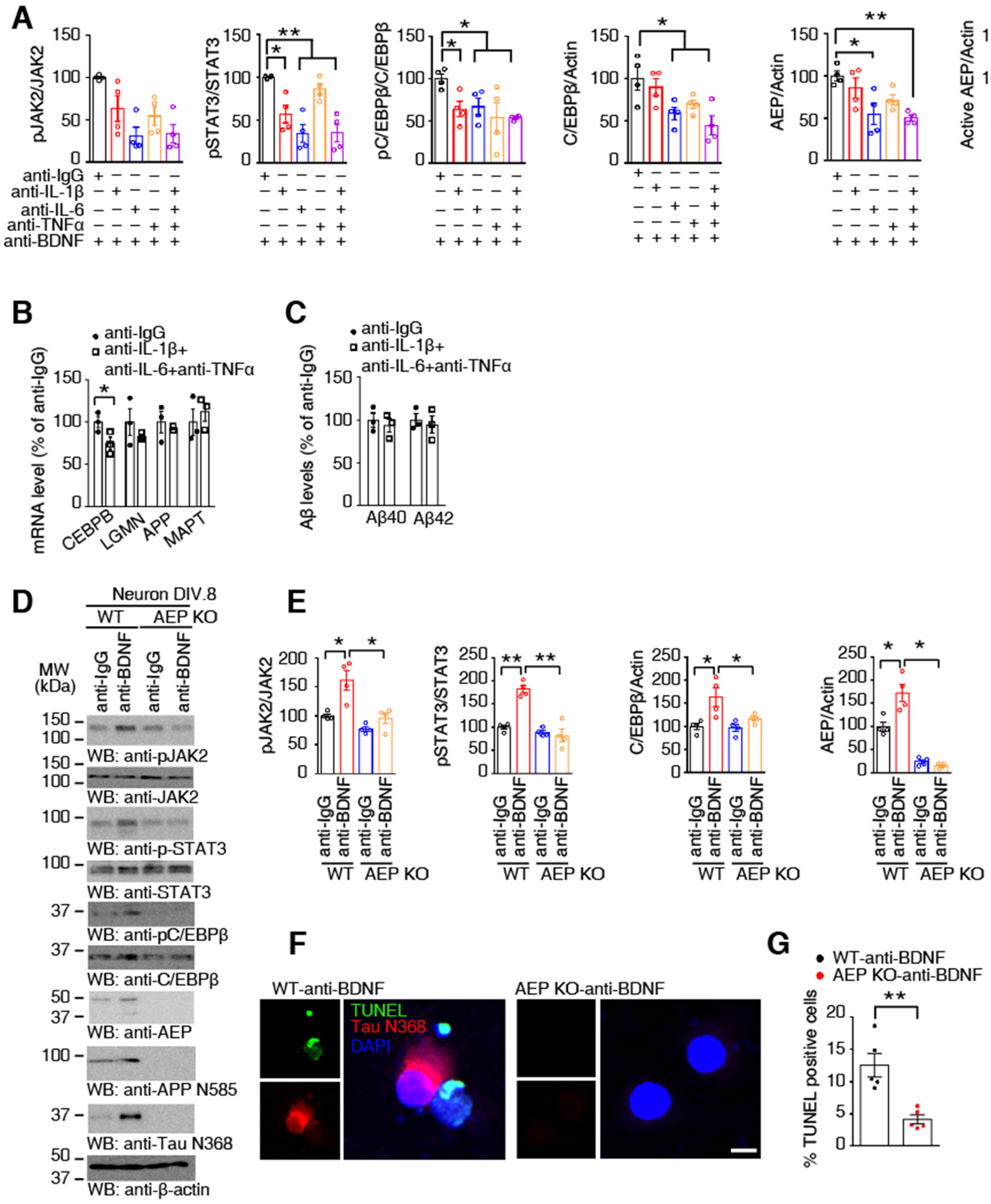

Fig. S2 Cytokine neutralization or AEP knockout inhibits JAK2/STAT3 and decreases the expression of C/EBPβ and delta-secretase. Related to Figure 2 and 3.

A Quantification of western blotting results from BDNF-deprived rat cortical neurons pretreated with various cytokine antibodies (n = 4, *P < 0.05, **P < 0.01, one-way ANOVA and Bonferroni's multiple comparison test).

B-C Effects of anti-inflammatory cytokine treatment on CEBPB, LGMN, APP, MAPT, and Aβ levels without anti-BDNF treatment (n = 3, *P < 0.05, one-way ANOVA and Bonferroni's multiple comparison test). **D** AEP

knockout prevents the cleavage of Tau and APP induced by BDNF deprivation. **E** Quantification of western blotting results in Fig. S3D (n = 4, *P < 0.05, **P < 0.01, one-way ANOVA and Bonferroni's multiple comparison test). **F-G** TUNEL and IF double-staining showing BDNF deprivation-induced neurotoxicity is repressed by AEP knockout. Scale bar, 10 μm. Quantification of TUNEL positive cells represents mean ± s.e.m. (n = 5 slides, **P < 0.01, unpaired t-test with Welch's correction).

Supplementary Figure 3

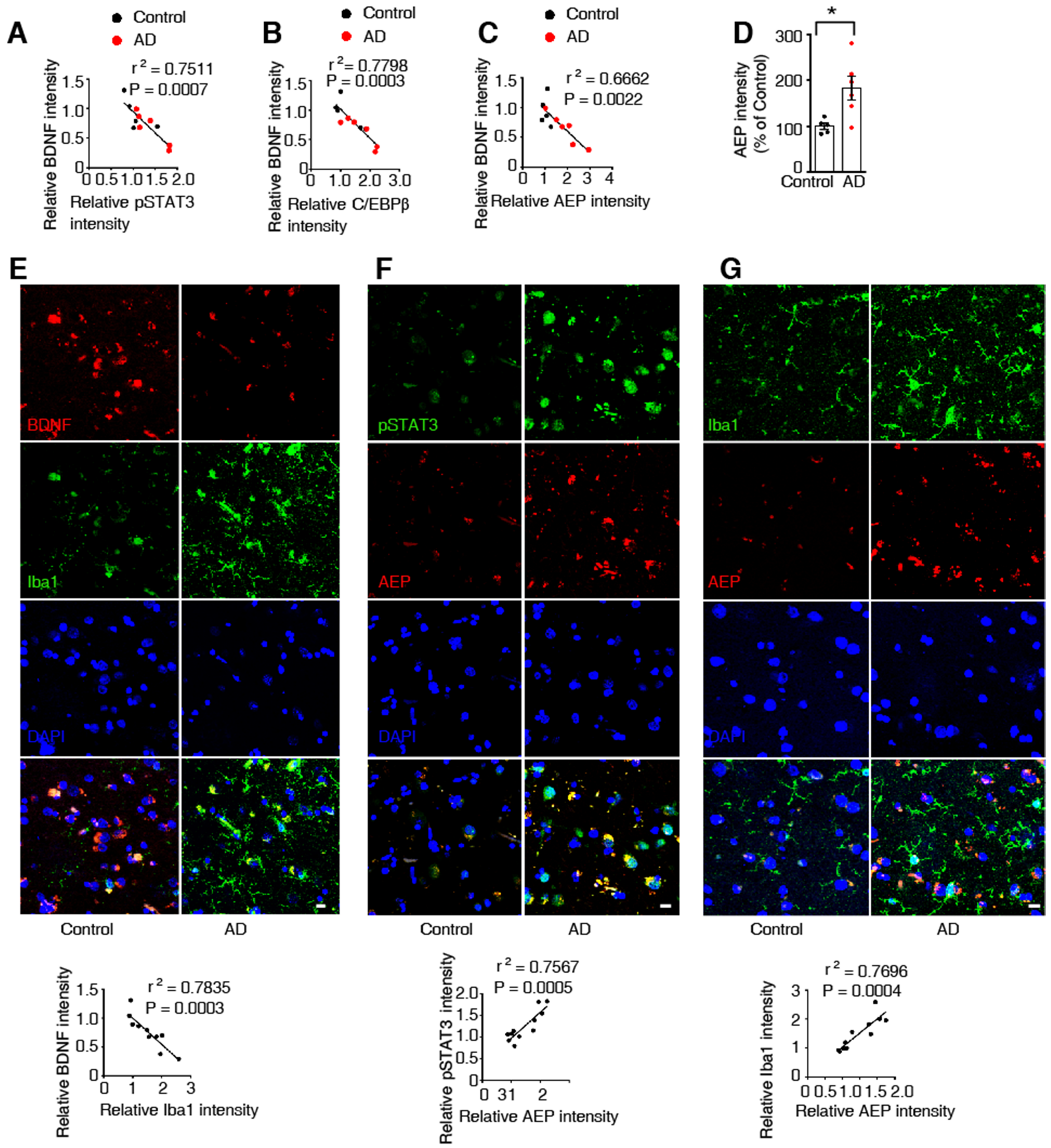

Fig. S3 Direct correlation between BDNF deficiency, neuroinflammation, and β-secretase upregulation in human AD brains. Related to Figure 4.

A-D Quantitative analysis of the fluorescent intensity signals revealing direct correlation between BDNF and each of p-STAT3, C/EBPβ and δ-secretase. The Spearman correlation coefficient r^2 and p value are shown. Quantification of AEP intensity represents mean ± s.e.m. (n=5 cases for control; n=6 cases for AD; *P < 0.05,

P < 0.01, unpaired t-test with Welch's correction). **E-G IF staining of BDNF/Iba1/pSTAT3/AEP in human hippocampus samples. IF data are representatives of 5 or 6 independent cases. Scale bar, 10 μm. Quantitative analysis of the fluorescent intensity signals revealing direct correlation between BDNF and Iba1, p-STAT3 and AEP, Iba1 and AEP. The Spearman correlation coefficient r^2 and p value are shown.

Supplementary Figure 4

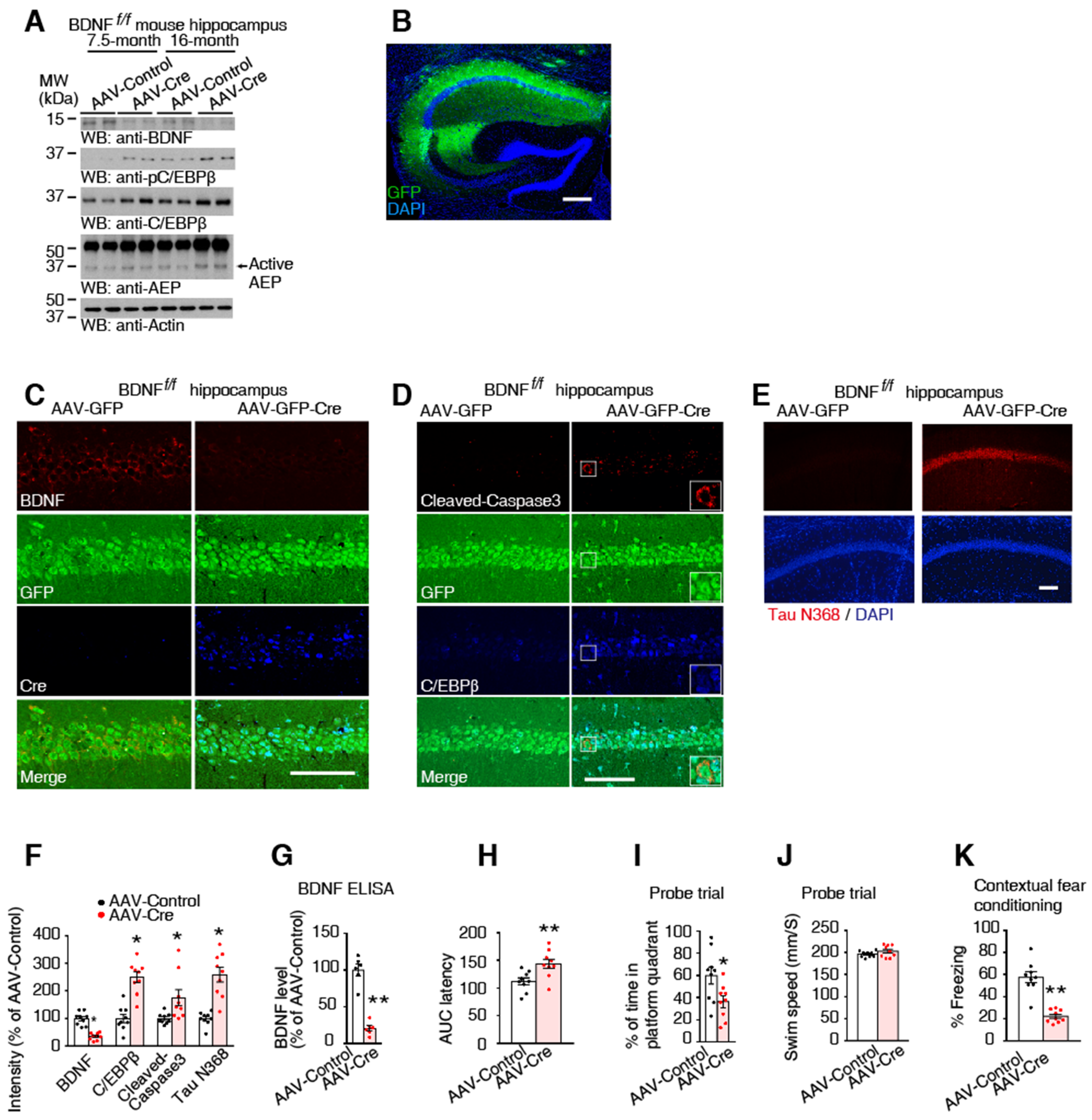

Fig. S4 BDNF knockout in hippocampus triggers murine Aβ and tau alterations. Related to Figure 5.

A BDNF knockout induced C/EBPβ/AEP activation in mice. **B** AAV-mediated GFP expression in BDNF$^{f/f}$ mice hippocampus. Scale bar, 200 μm. **C-E** IF staining of BDNF and Cre, cleaved-Caspase3 and C/EBPβ and Tau N368 in BDNF$^{f/f}$ mice hippocampal CA1 transduced with AAV-GFP or AAV-Cre. Scale bar, 100 μm. **F** Quantification of fluorescent intensity represent mean ± s.e.m. (n = 9 sections from 3 mice, *P < 0.05, unpaired t-test with Welch's correction). **G** Relative level of BDNF by ELISA (mean ± s.e.m.; n = 5 mice per group;

P<0.01, unpaired t-test with Welch's correction).** **H** Integrated latency (AUC) for mice (mean ± s.e.m.; n = 9 mice per group; **P<0.01, unpaired t-test with Welch's correction)**.** **I** Morris Water Maze analysis. BDNF knockout impaired memory (mean ± s.e.m.; n = 8-9 mice per group; *P< 0.05, unpaired t-test with Welch's correction). **J** The swim speed of mice in MWM test remained comparable (mean ± s.e.m.; n = 9 mice per group, unpaired t-test with Welch's correction). **K** Fear conditioning test. Contextual fear conditioning was reduced in BDNF knockout mice (mean ± s.e.m.; n = 9 mice per group; **P<0.01, unpaired t-test with Welch's correction)**.**

Supplementary Figure 5

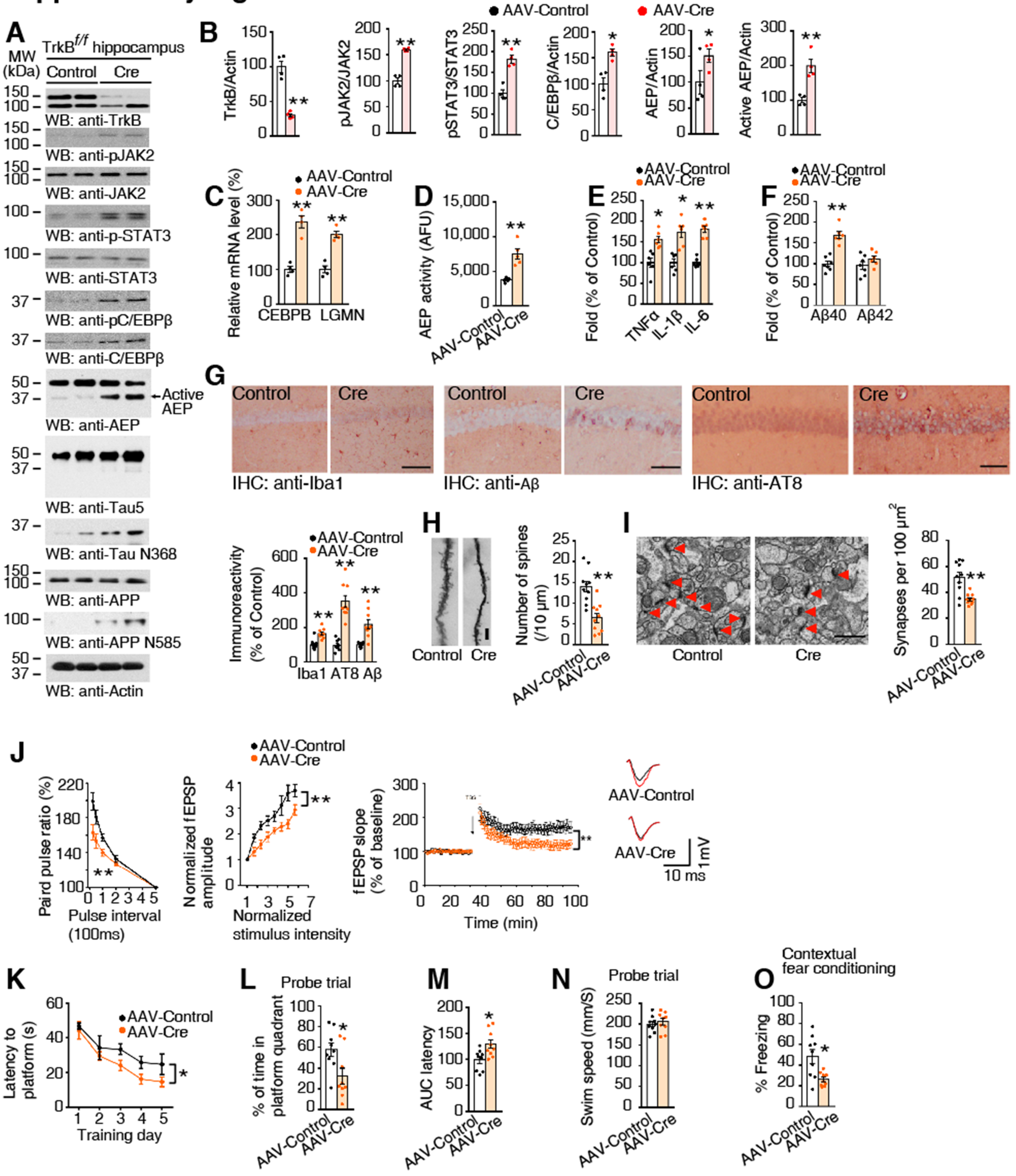

Fig. S5 Knockout of TrkB activates the JAK2/STAT3 pathway and induces murine Aβ and tau alterations. Related to Figure 5.

A TrkB knockout activates JAK2/STAT3 and increases the expression of C/EBPβ and δ-secretase. Immunoblotting was conducted from 16-month-old TrkB$^{f/f}$ mice hippocampus injected with AAV-Cre or AAV-GFP. Western blot data are representative of three independent experiments. **B** Quantification of western

blotting results in Fig. S5A (n = 4 mice, *P < 0.05, **P < 0.01, unpaired t-test with Welch's correction). **C** Quantitative RT-PCR analysis of CEBPB and LGMN mRNA levels in hippocampus with TrkB knockout. Data represent mean ± s.e.m. (n = 4, **P < 0.01, unpaired t-test with Welch's correction).**D** TrkB knockout increases δ-secretase enzymatic activities. Data represent mean ± s.e.m. (n = 5, *P < 0.05, unpaired t-test with Welch's correction). **E** TrkB knockout stimulates inflammatory cytokine production. Data represent mean ± s.e.m. (n = 6, *P < 0.05, **P < 0.01, unpaired t-test with Welch's correction). **F** TrkB knockout increases Aβ production. Quantification of Aβ40 and Aβ42 levels by ELISA represents mean ± s.e.m. (n = 6-7 mice, *P < 0.05, **P < 0.01, unpaired t-test with Welch's correction). **G** IHC of anti-Iba1, anti-Aβ and anti-AT8 showing that TrkB knockout promotes mouse Aβ and tau alterations. Scale bar, 50 µm. Data shown as mean ± s.e.m. of 10 sections from three mice (*P < 0.05, unpaired t-test with Welch's correction). **H** TrkB knockout decreases the dendritic spine density. Golgi staining was conducted on brain sections from apical dendritic layer of the CA1 region. Scale bar, 5 µm. Data represent mean ± s.e.m. of 10 sections from 3 mice in each group. (*P < 0.05, unpaired t-test with Welch's correction). **I** EM analysis demonstrating that the synapses are decreased in the TrkB knockout hippocampus. Scale bar, 1 µm. Data represent mean ± s.e.m. of 10 sections from 3 mice in each group. (*P < 0.05, unpaired t-test with Welch's correction). **J** Electrophysiology analysis. TrkB knockout leaded to LTP defects. The ratio of paired pulses in different groups (mean ± s.e.m.; n = 6 in each group; **P < 0.01, two-way ANOVA) (left). Input-output curve (middle) represent mean ± s.e.m. of 6 mice per group (**P < 0.01, two-way ANOVA). LTP of fEPSPs (mean ± s.e.m.; n = 6 in each group; **P < 0.01, two-way ANOVA) (right). Shown traces are representative fEPSPs recorded before (black) and 60 minutes after (red) TBS. **K-M** Morris Water Maze analysis. TrkB knockout impaired the learning and memory (mean ± s.e.m.; n = 9 mice per group; *P< 0.05, two-way ANOVA for **K** and unpaired t-test with Welch's correction for **L** and **M**). **N** The swim speed of mice in MWM test remained comparable (mean ± s.e.m.; n = 9 mice per group, unpaired t-test with Welch's

correction). **O** Fear condition test. Contextual fear conditioning was reduced in TrkB knockout mice (mean ± s.e.m.; n = 9 mice per group; *P<0.05, unpaired t-test with Welch's correction).

Supplementary Figure 6

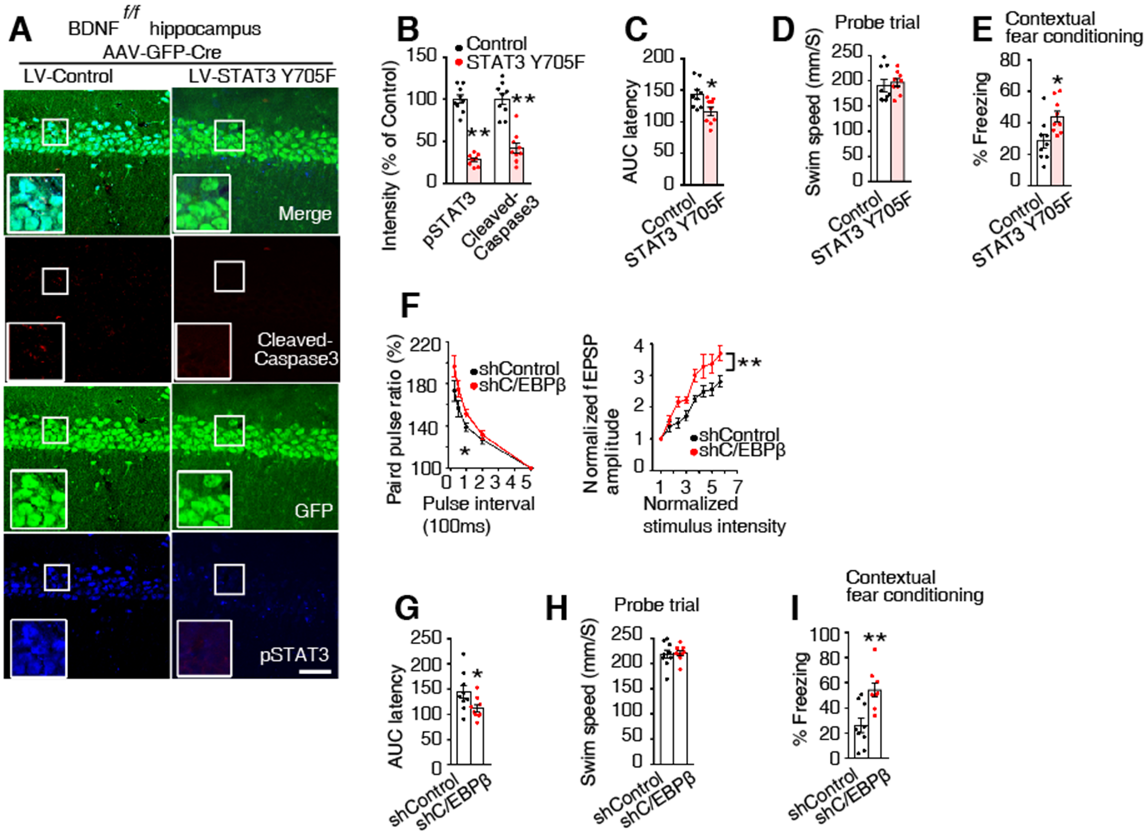

Fig. S6 STAT3 Y705F mutant expression rescues BDNF depletion-mediated neuronal apoptosis and cognitive deficits. Related to Figure 6 and 7.

A IF staining of cleaved-Caspase 3 and pSTAT3 in BDNF flox/flox mice hippocampal CA1 injected with AAV-Cre + LV-Control or AAV-Cre + LV-STAT3 Y705F. Scale bar, 50 µm. **B** Quantification of fluorescent intensity represents mean ± s.e.m. (n = 9 sections from 3 mice, *P < 0.05, unpaired t-test with Welch's correction). **C** Integrated latency (AUC) for mice (mean ± s.e.m.; n = 9 mice per group; *P < 0.05, unpaired t-test with Welch's correction)**. D** The swim speed of mice in MWM test remained comparable (mean ± s.e.m.; n = 9 mice per group, unpaired t-test with Welch's correction)**. E** Fear condition test. Contextual fear conditioning was reduced in BDNF knockout mice (mean ± s.e.m.; n = 9 mice per group; *P<0.05, unpaired t-test with Welch's correction)**. F** Electrophysiology analysis. The ratio of paired pulses (lift) in different groups (mean ± s.e.m.; n = 6 in each group; *P < 0.05, two-way ANOVA and Bonferroni's post hoc test). Input-output

curves (right) represent mean ± s.e.m. of 6 mice per group (*$P < 0.05$, two-way ANOVA and Bonferroni's post hoc test). **G** Integrated latency (AUC) for mice (mean ± s.e.m.; n = 9 mice per group; *$P < 0.05$, unpaired t-test with Welch's correction)**.** **H** The swim speed of mice in MWM test remained comparable (mean ± s.e.m.; n = 9 mice per group, unpaired t-test with Welch's correction)**.** **I** Fear conditioning test. Contextual fear conditioning was reduced in BDNF knockout mice (mean ± s.e.m.; n = 9 mice per group; **$P < 0.01$, unpaired t-test with Welch's correction)**.**

Supplementary Figure 7

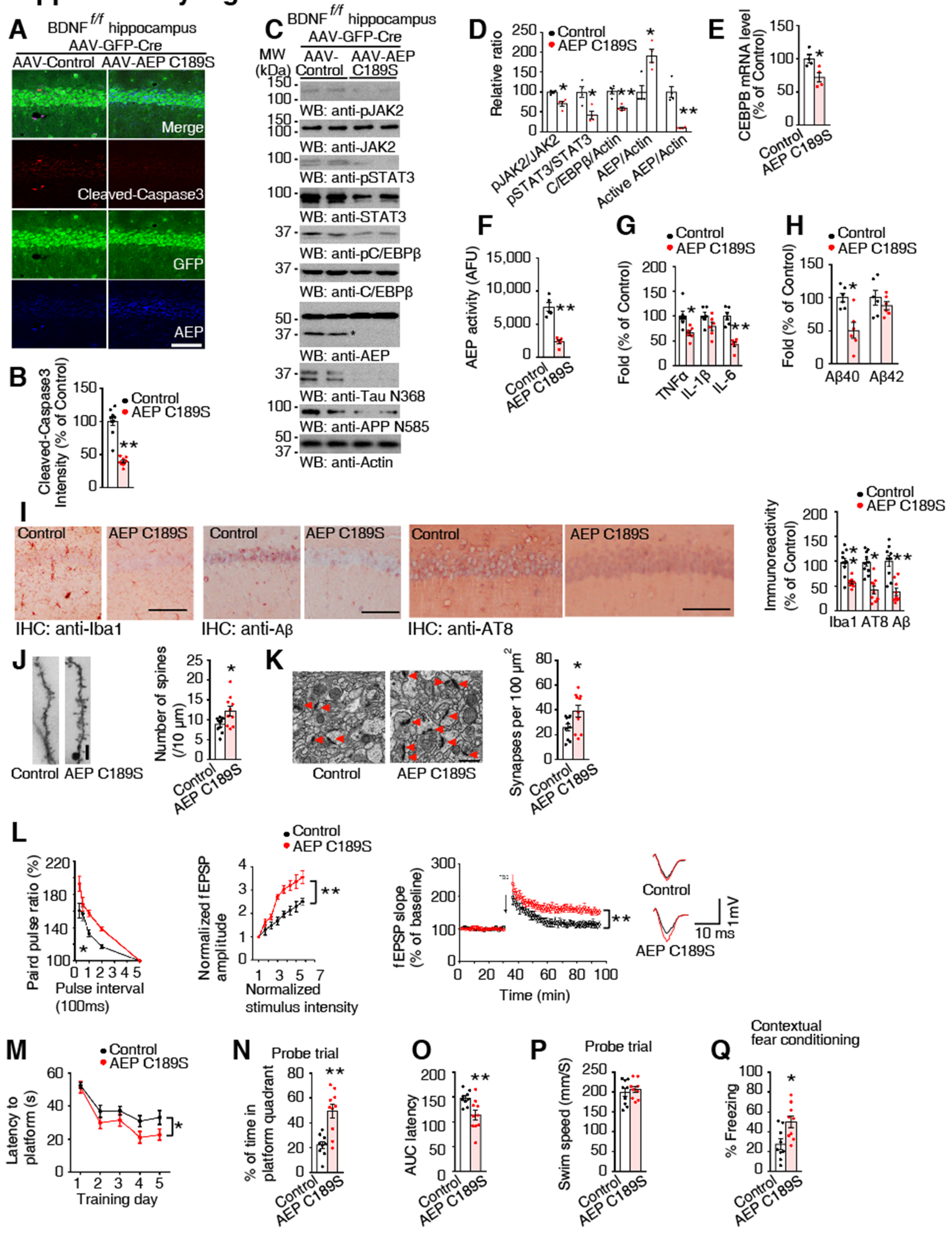

Fig. S7 Inhibition of delta-secretase represses BDNF depletion-induced neuronal apoptosis and murine Aβ and tau alterations. Related to Figure 7.

A-B IF staining of cleaved-Caspase 3 and AEP in BDNF flox/flox mice hippocampal CA1 injected with AAV-Cre + AAV-Control or AAV-Cre +AAV-AEP C189S. Scale bar, 50 μm. Quantification of fluorescent intensity represents mean ± s.e.m. (n = 9 sections from 3 mice, *P < 0.05, unpaired t-test with Welch's correction). **C** AAV-AEP C189S expression prevents δ-secretase activation in the BDNF-depleted hippocampus. Immunoblotting was conducted from 16-month-old BDNF flox/flox mice hippocampus co-injected with AAV-Cre and AAV-Control or AAV-Cre and AAV-AEP C189S. Western blot data are representative of three independent experiments. *Active AEP. **D** Quantification of western blotting results (n = 4 mice, *P < 0.05, **P < 0.01, unpaired t-test with Welch's correction). **E** Quantitative RT-PCR analysis of CEBPB mRNA levels in $BDNF^{f/f}$ mice hippocampus. Data represent mean ± s.e.m. of four mice (**P < 0.01, unpaired t-test with Welch's correction). **F** BDNF knockout-induced activation of δ-secretase is lessened by AEP C189S expression. Data represent mean ± s.e.m. of five mice (*P < 0.05, unpaired t-test with Welch's correction). **G** Relative cytokine levels measured by ELISA of hippocampus lysates. Data represent mean ± s.e.m. of six samples per group (*P < 0.05, **P < 0.01, unpaired t-test with Welch's correction). **H** Inhibition of δ-secretase represses Aβ production induced by BDNF knockout. Quantification of Aβ40 and Aβ42 levels by ELISA represents mean ± s.e.m. (n = 6 mice,*P < 0.05, **P < 0.01, unpaired t-test with Welch's correction). **I** Immunohistochemistry (IHC) of anti-Iba1, anti-Aβ and anti-AT8 in hippocampal CA1. Scale bar, 50 μm. Data shown as mean ± s.e.m. of 6 sections from three mice (*P < 0.05, unpaired t-test with Welch's correction).**J** AEP C189S increases the dendritic spine density. Golgi staining was conducted on brain sections from apical dendritic layer of the CA1 region. Scale bar, 5 μm. Data on the right represent mean ± s.e.m. of 10 sections from 3 mice in each group. (*P < 0.05, unpaired t-test with Welch's correction). **K** EM analysis. Scale bar, 1 μm. Data represent mean ± s.e.m. of 10 sections from 3 mice in each group. (*P < 0.05, unpaired t-test with Welch's correction). **L** Electrophysiology analysis. AEP C189S rescued LTP defects. The ratio of paired pulses in different groups

(mean ± s.e.m.; n = 6 in each group; *P < 0.05, two-way ANOVA and Bonferroni's post hoc test) (left). Input-output curve (middle) represent mean ± s.e.m. of 6 mice per group (**P < 0.01, two-way ANOVA and Bonferroni's post hoc test). LTP of fEPSPs (mean ± s.e.m.; n = 6 mice in each group; **P < 0.01, two-way ANOVA and Bonferroni's post hoc test). Shown traces are representative fEPSPs recorded before (black) and 60 minutes after (red) TBS. **M-Q** Morris Water Maze and Fear conditioning. AEP C189S ameliorated the learning and memory impairment (mean ± s.e.m.; n = 9 mice per group; *P< 0.05, **P< 0.01, two-way ANOVA and Bonferroni's post hoc test for **n** and unpaired t-test with Welch's correction for **o-r**).
