# Supplementary material for: Deficiency in BDNF/TrkB Neurotrophic Activity Stimulates δ-Secretase by Upregulating C/EBPβ in Alzheimer’s Disease
Source: Cell Rep. Author manuscript; Available in PMC 2019 Aug 6. (PMC6684282; doi:10.1016/j.celrep.2019.06.054)
Supplement: 2 [file NIHMS1535616-supplement-2.pdf]

Cell Reports

Article

# Deficiency in BDNF/TrkB Neurotrophic Activity Stimulates δ-Secretase by Upregulating C/EBPβ in Alzheimer's Disease

## Graphical Abstract

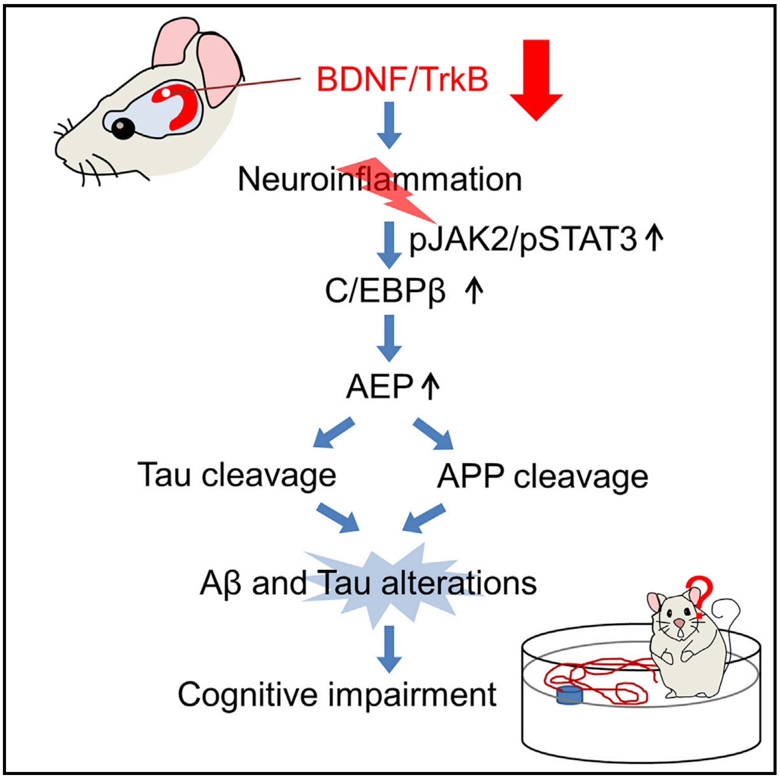


## Highlights

- Deprivation of BDNF increases inflammatory cytokines
- Deprivation of BDNF upregulates C/EBPβ by JAK2/STAT3 pathway
- BDNF knockout increases expression of AEP by C/EBPβ
- Inhibition of JAK2/STAT3/C/EBPβ/AEP prevents BDNF-depletion-mediated pathology

## Authors

Zhi-Hao Wang, Jie Xiang, Xia Liu, ..., Shengxi Wu, Jian-Zhi Wang, Keqiang Ye

## Correspondence

wangjz@mails.tjmu.edu.cn (J.-Z.W.), kye@emory.edu (K.Y.)

## In Brief

Deficient BDNF/TrkB activity underlies AD pathogenesis. Wang et al. report that deprivation of BDNF/TrkB increases inflammatory cytokines and activates the JAK2/STAT3 pathway, resulting in the upregulation of C/EBPβ/AEP signaling. Reduction of BDNF is inversely coupled with the aforementioned pathway in AD brains. Inhibition of JAK2/STAT3/C/EBPβ/AEP prevents BDNF-depletion-mediated pathology.

Wang et al., 2019, Cell Reports 28, 655–669
July 16, 2019 © 2019 The Authors.
https://doi.org/10.1016/j.celrep.2019.06.054

CellPress

OPEN ACCESS
CellPress

# Deficiency in BDNF/TrkB Neurotrophic Activity Stimulates δ-Secretase by Upregulating C/EBPβ in Alzheimer's Disease

Zhi-Hao Wang,[1,2] Jie Xiang,[1,4] Xia Liu,[1] Shan Ping Yu,[5] Fredric P. Manfredsson,[6] Ivette M. Sandoval,[6] Shengxi Wu,[4] Jian-Zhi Wang,[2,3,*] and Keqiang Ye[1,7,*]

[1]Department of Pathology and Laboratory Medicine, Emory University School of Medicine, Atlanta, GA 30322, USA
[2]Department of Pathophysiology, Key Laboratory of Ministry of Education of Neurological Diseases, Tongji Medical College, Huazhong University of Science and Technology, Wuhan, Hubei 430030, China
[3]Co-innovation Center of Neuroregeneration, Nantong, Jiangsu 226001, China
[4]Department of Neurobiology, Fourth Military Medical University, Xi'an, Shaanxi 710032, China
[5]Department of Anesthesiology, Emory University School of Medicine, Atlanta, GA 30322, USA
[6]Department of Translational Science & Molecular Medicine, Michigan State University, 333 Bostwick Ave. NE, Grand Rapids, MI 49503, USA
[7]Lead Contact
*Correspondence: wangjz@mails.tjmu.edu.cn (J.-Z.W.), kye@emory.edu (K.Y.)
https://doi.org/10.1016/j.celrep.2019.06.054

## SUMMARY

**BDNF/TrkB neurotrophic signaling regulates neuronal development, differentiation, and survival, and deficient BDNF/TrkB activity underlies neurodegeneration in Alzheimer's disease (AD). However, exactly how BDNF/TrkB participates in AD pathology remains unclear. Here, we show that deprivation of BDNF/TrkB increases inflammatory cytokines and activates the JAK2/STAT3 pathway, resulting in the upregulation of transcription factor C/EBPβ. This, in turn, results in increased expression of δ-secretase, leading to both APP and Tau fragmentation by δ-secretase and neuronal loss, which can be blocked by expression of STAT3 Y705F, knockdown of C/EBPβ, or the δ-secretase enzymatic-dead C189S mutant. Inhibition of this pathological cascade can also rescue impaired synaptic plasticity and cognitive dysfunctions. Importantly, reduction in BDNF/TrkB neurotrophic signaling is inversely coupled with an increase in JAK2/STAT3, C/EBPβ, and δ-secretase escalation in human AD brains. Therefore, our findings provide a mechanistic link between BDNF/TrkB reduction, C/EBPβ upregulation, δ-secretase activity, and Aβ and Tau alterations in murine brains.**

## INTRODUCTION

Neurotrophins are growth factors that regulate neuronal development, differentiation, and survival in both the peripheral nervous system and CNS. Neurotrophins consist of four structure-related proteins, nerve growth factor (NGF), brain-derived neurotrophic factor (BDNF), neurotrophin-3 (NT-3), and neurotrophin-4 (NT-4/5) (Huang and Reichardt, 2003). It has been documented that BDNF expression is reduced in the brains of Alzheimer's disease (AD) patients (Connor et al., 1997; Ferrer et al., 1999; Peng et al., 2009; Phillips et al., 1991). AD is characterized by the accumulation of β-amyloid peptide (Aβ) within the brain along with hyperphosphorylated and cleaved forms of the microtubule-associated protein Tau. The physiopathology of AD is poorly understood. Nevertheless, it is known that impaired metabolism of the amyloid β precursor protein (APP) and abnormal Tau protein phosphorylation lead to the formation of neuritic plaques and neurofibrillary tangles (NFTs), respectively. These events result in neurodegeneration and the clinical expression of dementia (Selkoe, 1996). Genetic, biochemical, and behavioral research suggests that generation of the neurotoxic Aβ peptide from sequential APP proteolysis is the crucial step in the development of AD (Ballatore et al., 2007). Accumulative evidence suggests that BDNF/TrkB signaling may be an important regulator of amyloidogenic processing. For instance, Aβ production is reduced by BDNF in primary neuronal cultures (Rohe et al., 2009), whereas it is elevated by BDNF deprivation (Matrone et al., 2008). Interestingly, neurons containing NFTs do not express BDNF, whereas neurons with a high degree of BDNF expression are devoid of tangles (Murer et al., 1999). Thus, BDNF may have a protective role against AD pathogenesis. The beneficial effect of BDNF administration has been shown to increase learning and memory in impaired animals (Ando et al., 2002), and studies in AD models show that BDNF has a neuroprotective effect against β-amyloid toxicity. BDNF gene delivery has, thus, been proposed as a potential therapeutic in AD (Arancibia et al., 2008; Kitiyanant et al., 2012).

Recently, we reported that δ-secretase, an asparagine endopeptidase (AEP, gene name *LGMN*), cleaves both APP and Tau in an age-dependent manner in mouse brains and human AD brains. Its enzymatic activity and expression level increase in an age-dependent manner. AEP cleaves APP at both the N373 and N585 residues in the ectodomain and facilitates Aβ production by decreasing the steric hindrance for BACE1. Depletion of δ-secretase significantly reduces Aβ production and senile plaque formation in 5XFAD mouse brains, leading to restoration of synaptic activity and cognitive function (Zhang et al., 2015). Additionally, δ-secretase cleaves Tau at N255 and N368 and abolishes its microtubule assembly activity, resulting


This is an open access article under the CC BY license (http://creativecommons.org/licenses/by/4.0/).

OPEN ACCESS
CellPress

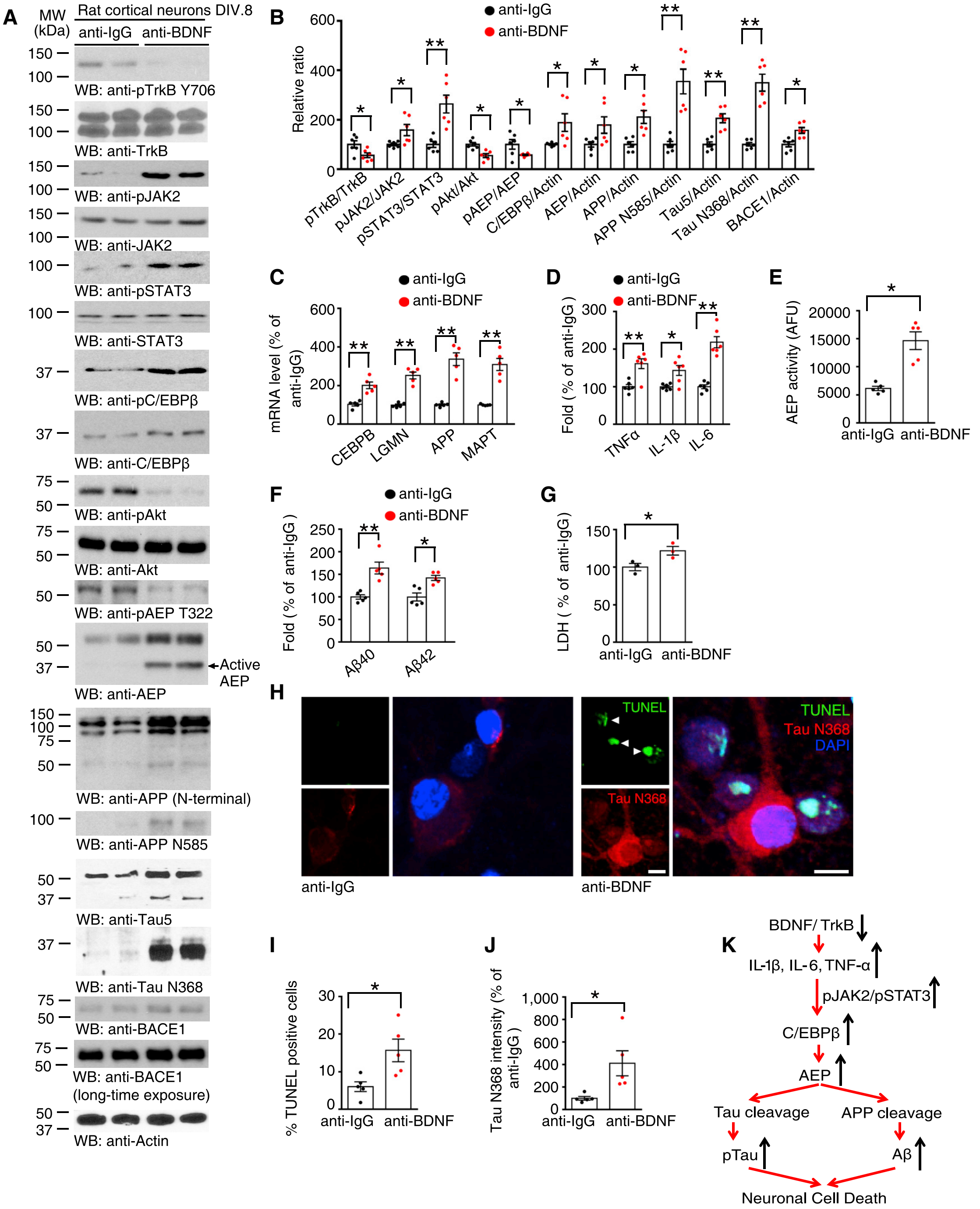


**Figure 1. Deprivation of BDNF Increases Cytokines and Activates the JAK2/STAT3 Pathway in Primary Neurons**

(A) BDNF deprivation activates JAK2/STAT3 and increases the expressions of C/EBPβ and δ-secretase. Immunoblotting was conducted from primary neurons after incubation with anti-BDNF or anti-IgG antibody. Data are representative of three independent experiments.

(B) Quantification of western blotting (n = 6 per group, *p < 0.05, **p < 0.01, unpaired t test with Welch's correction).

(C) qRT-PCR analysis of C/EBPβ, LGMN, APP, and MAPT mRNA levels in neurons treated with anti-BDNF or anti-IgG antibody. Data represent mean ± SEM of 5 independent experiments (**p < 0.01, unpaired t test with Welch's correction).

(D) ELISA of whole-cell lysates. Data represent mean ± SEM of 6 samples per group from 3 independent experiments (*p < 0.05, **p < 0.01, unpaired t test with Welch's correction).

(E) BDNF deprivation increases δ-secretase enzymatic activity. Data represent mean ± SEM (n = 5, *p < 0.05, unpaired t test with Welch's correction).

(F) BDNF deprivation stimulates Aβ production. Quantification of Aβ40 and Aβ42 levels by ELISA represents mean ± SEM (n = 5, *p < 0.05, **p < 0.01, unpaired t test with Welch's correction).

*(legend continued on next page)*

in its aggregation and NFT formation. Notably, the cleaved Tau 1-368 fragment is neurotoxic. Deletion of δ-secretase from Tau P301S mice reverses synaptic defects and cognitive dysfunction (Zhang et al., 2014). Hence, δ-secretase is a crucial component in AD onset and progression. Most recently, we showed that Akt phosphorylates δ-secretase on T322, repressing its proteolytic activation (Wang et al., 2018b). Previously, we have reported that δ-secretase cleaves SET (also called PHAPII, TAF-Iβ, and $I_2^{PP2A}$), which possesses dual roles in inhibiting both PP2A phosphatase (Li et al., 1996) and DNase (Fan et al., 2003), triggering neuronal cell death (Liu et al., 2008). SET cleavage by δ-secretase also induces Tau hyperphosphorylation, by blocking PP2A (Arnaud et al., 2011). We also identified that an age-dependent transcription factor, C/EBPβ (CCAAT-enhancer binding protein β), controls δ-secretase expression in the brain, promoting AD pathology (Wang et al., 2018a). In addition to promoting the production of inflammatory mediators, C/EBP family members are themselves induced by the classical pro-inflammatory triad of IL-1β (interleukin-1β), IL-6, and tumor necrosis factor alpha (TNF-α) (Künzi and Pitha, 1996; Magalini et al., 1995; Poli, 1998; Wedel and Ziegler-Heitbrock, 1995), all of which are significantly increased in pathologically impacted regions of the AD brain (Akiyama et al., 2000). One of the hallmarks of AD is chronic neuroinflammation mediated by astrocytes and microglial cells, most likely induced by the formation of Aβ deposits. Importantly, Aβ and IL-6 additively increase C/EBPβ activity (Strohmeyer et al., 2014). Hence, there is a feedback loop between amyloid and neuroinflammation through activating C/EBPβ in microglia or astrocytes.

The non-receptor Janus kinase 2/signal transducer and activator 3 of transcription (JAK2/STAT3) signaling pathway are vital components of neuro-immune responses (Wang et al., 2014), where they are regulated by various pro-inflammatory cytokines. A substantial number of key class I cytokine receptors utilize JAK2/STATs for signaling (O'Sullivan et al., 2007). The cytokine possesses multiple binding sites for its receptor, and upon binding, these facilitate dimerization or multimerization of their cognate class 1 cytokine receptors. This then results in close apposition of the kinase domains of two membrane proximal receptor-associated JAK2s within the cells and, hence, their transactivation (Waters and Brooks, 2015). For instance, IL-6, through the IL-6 receptor, triggers the phosphorylation and activation of JAK2 (Y1007/1008) and STAT3 (Y705) (Berishaj et al., 2007; Liu et al., 2011; Ni et al., 2004). STATs are important cellular mediators of cytokine receptor signaling and regulate transcription of target genes. JAK2/STAT3 activation also initiates the transcription and expression of several specific inflammatory genes, such as TNF-α, IL-6, and IL-1β, resulting in the excessive accumulation of the corresponding inflammatory mediators (Agrawal et al., 2011). Interestingly, STAT3 also acts as a transcription factor for C/EBPβ by binding the distal region of the C/EBPβ promoter (Zhang et al., 2011). In the current work, we provide extensive evidence demonstrating that BDNF/TrkB neurotrophic reduction elicits the secretion of inflammatory cytokines that activates the JAK2/STAT3 pathway, which mediates C/EBPβ mRNA transcription. Subsequently, C/EBPβ elevates δ-secretase expression, resulting in cleavage of both APP and Tau and promoting the onset of AD pathology. Our findings establish a signaling pathway, going from JAK2/STAT3 to C/EBPβ to δ-secretase, delineating the molecular mechanisms of how BDNF/TrkB deficiency contributes to AD pathogenesis.

## RESULTS

### BDNF Deprivation Increases Levels of Inflammatory Cytokines That Activate JAK2/STAT3, Elevating C/EBPβ Expression

To inhibit endogenous BDNF secreted from neurons, we applied a specific BDNF antibody to rat primary neurons. The specificity of this antibody has previously been demonstrated (Matrone et al., 2008), and a recent study uses it in rat brain (Lambert et al., 2017). Compared to control anti-immunoglobulin G (IgG), the anti-BDNF antibody strongly inhibited p-TrkB Y706 in these neurons, and this disruption of the BDNF/TrkB signaling pathways robustly triggered both JAK2 and STAT3 activation, as indicated by tyrosine phosphorylation. As a result, levels of the downstream transcriptive target of STAT3, C/EBPβ, were elevated. This, in turn, resulted in an increase in expression of δ-secretase, a transcription target of C/EBPβ (Figure 1A, top to 8th panels; Figure 1B). We recently demonstrated that δ-secretase is a substrate of Akt and that phosphorylation of T322 on δ-secretase by Akt blocks its enzymatic activation (Wang et al., 2018b). Thus, reduction in Akt-mediated T322 phosphorylation due to BDNF neutralization provoked δ-secretase activation. Indeed, both APP and Tau were upregulated and truncated by active δ-secretase, as seen by the presence of immunoreactivity specific to both APP N585 and Tau N368 (Figure 1A, 13th–bottom panels; Figure 1B). δ-secretase cleavage and its relation to APP involve an intimate coupling of β- and δ-secretase. BDNF deprivation also upregulated BACE1 protein levels (Figure 1A, 18th–bottom panels; Figure 1B). Given that numerous BACE1 antibodies detect unspecific bands, the identity of the relevant protein bands were ascertained by a knockout approach (Hu et al., 2018) (Figure S1A). qRT-PCR analysis revealed that BDNF deprivation increased transcription of C/EBPβ, LGMN, APP, and microtubule associated protein Tau (MAPT) (Figure 1C). Moreover, the production of inflammatory cytokines IL-1β, IL-6, and TNFα was increased upon BDNF deprivation (Figure 1D). Interestingly, BDNF inhibition in primary neurons resulted in δ-secretase activation (Figure 1E). δ-Secretase proteolytic cleavage of APP N585 resulted in elevated Aβ40 and 42 formation (Figure 1F). The rat Aβ ELISA was validated in rat neurons treated with β- or

(G) LDH assay showing that BDNF deprivation promotes cell death. Data shown as mean ± SEM of 3 independent experiments (*$p < 0.05$, unpaired t test with Welch's correction).

(H–J) TUNEL and immunofluorescence (IF) double-labeling assay (H). Scale bar, 10 μm. Quantifications of TUNEL positive cells (I) and Tau N368 level (J) represent mean ± SEM (n = 6, *$p < 0.05$, unpaired t test with Welch's correction). Scale bars, 10 μm.

(K) A schematic diagram showing directionality of gene and encoded protein changes.

See also Figure S1.

γ-secretase inhibitors (Figure S1B). Extensive neuronal cell death was found in anti-BDNF-treated neurons (Figure 1G). This effect was validated by TUNEL staining: anti-BDNF triggered demonstrable neuronal apoptosis and immunofluorescent (IF) co-staining revealed that this effect correlated with Tau N368 cleavage (Figures 1H–1J; Figure S1C). Accordingly, we hypothesized that deprivation of BDNF in the brain increases inflammatory cytokines and activates the JAK2/STAT3 pathway, leading to the upregulation of transcription factor C/EBPβ. This, in turn, results in increased expression of δ-secretase, leading to both APP and Tau fragmentation and neuronal loss (Figure 1K).

To further assess the biological effects of BDNF/TrkB deprivation, we prepared primary neurons from transgenic homozygous BDNF-floxed mice (Rios et al., 2001) (*Bdnf*$^{tm3Jae}$/J, referred herein as BDNF$^{f/f}$) neonatal pups and knocked out the BDNF gene by adeno-associated virus (AAV)-Cre virus. As shown by immunoblotting, BDNF depletion triggered JAK2/STAT3 activation (Figures S1D and S1E). Subsequently, expressions of the STAT3 responsive gene CCAAT/enhancer binding protein (C/EBP) beta (CEBPB) and its downstream target *LGMN* increased (Figures S1D and S1E). As was seen with anti-BDNF treatment, CEBPB, LGMN, APP, and MAPT transcripts were significantly elevated following BDNF removal (Figure S1F). The enzymatic assay indicated that BDNF knockout elicited δ-secretase activation (Figure S1G). Similarly, short hairpin RNA (shRNA)-mediated knockdown of TrkB from primary neurons further supported these findings. TrkB depletion repressed p-Akt, decreased p-AEP T322 and escalated the proteolytic maturation of AEP (Figures S1H–S1J). Thus, inhibition of BDNF signaling (by BDNF or TrkB deletion) results in upregulation of pro-inflammatory cytokines, associated with JAK2/STAT3 signaling activation and subsequent escalation of C/EBPβ and its downstream target δ-secretase. Consequently, active δ-secretase cleaves both APP and Tau, resulting in Aβ augmentation and neuronal cell death.

## Neutralization of Cytokines Inhibits BDNF-Deprivation-Mediated STAT3 Activation and C/EBPβ Expression

To assess whether JAK2/STAT3 pathway activation as a result of BDNF deprivation is mediated by pro-inflammatory cytokines, we pretreated primary neurons with various antibodies separately, or in combination, prior to treatment with the anti-BDNF antibody. ELISA validation showed that these cytokines were reduced (Figure 2A). Incubation with antibodies against IL-1β or IL-6 repressed p-JAK2 and p-STAT3 content upon BDNF deprivation, as compared to the anti-IgG control. The maximal effect for JAK2 inhibition occurred when all these 3 cytokines were neutralized, which also resulted in a complete inhibition of STAT3 phosphorylation. Levels of the downstream readout C/EBPβ corresponded with JAK2/STAT3 activation levels (Figure 2B, top–6$^{th}$ panel, Figure S2A). Moreover, δ-secretase activation, and the subsequent truncation of APP N585 and Tau N368, correlated with JAK2/STAT3 activation (Figure 2B, 7$^{th}$–bottom panel; Figure S2A). RT-PCR showed that C/EBPβ expression was reduced as a result of neutralizing each pro-inflammatory cytokine, with anti-IL-6 producing the strongest effect, similar to a combination of all three antibodies (Figure 2C). This suggests that IL-6 might be the most crucial factor for mediating C/EBPβ transcription among these cytokines. The effects on the other three genes, including LGMN, APP, and MAPT, were similar (Figure 2C). A comparable pattern of enzymatic activity of δ-secretase was found in these treatments, in alignment with *LGMN* transcription levels (Figure 2D). Blocking the pro-inflammatory cytokines by the antibodies significantly repressed the increased production of both Aβ40 and Aβ42 induced by BDNF deprivation (Figure 2E). Antagonizing the inflammatory cytokines induced by BDNF deprivation also reduced neuronal cell death, as shown by lactate dehydrogenase (LDH) assay and TUNEL staining (Figures 2F–2I). Neutralizing pro-inflammatory cytokines without anti-BDNF treatment decreased C/EBPβ transcription level, with no significant effects on LGMN, APP, MAPT, and Aβ levels (Figures S2B and S2C). Therefore, BDNF-deficiency-triggered C/EBPβ activation and downstream gene expression, as well as neural cytotoxicity, might be mediated by the secreted pro-inflammatory cytokines.

## Blockade of STAT3 or C/EBPβ Rescues BDNF-Depletion-Induced δ-Secretase Activation

JAK2-mediated phosphorylation of STAT3 at tyrosine 705 results in dimerization and translocation from the cytoplasm to the nucleus, where STAT3 binds to specific DNA elements to regulate transcription (Yu et al., 2007). STAT3 Y705F may act as a dominant-negative effector to compete with endogenous STAT3 for activation (Kaptein et al., 1996). To better understand the role of STAT3 in BDNF-deprivation-induced events, we used lentiviral vectors to express the unphosphorylatable STAT3 Y705F mutant in primary neuronal cultures, followed by treatment with anti-BDNF IgG. BDNF depletion resulted in increased C/EBPβ expression, which was mitigated by STAT3 Y705F expression, and LGMN expression correlated with C/EBPβ levels. As a result, APP N585 and Tau N368 cleavage by δ-secretase was attenuated (Figures 3A and 3B). The augmented CEBPB and LGMN mRNA levels provoked by BDNF deprivation were significantly repressed in cultures expressing STAT3 Y705F (Figure 3C). Levels of cytokines triggered by BDNF deprivation were not significantly affected by STAT3 Y705F (Figure 3D). Consistent with the reduced expression of LGMN, δ-secretase enzymatic activities were substantially reduced (Figure 3E). Interestingly, the generation of Aβ40 but not Aβ42 induced by BDNF deprivation was suppressed by STAT3 Y705F expression (Figure 3F). Presumably, it is because that Aβ42 is produced at much lower levels than Aβ40 (Golde, 2003) (as in most non-Tg AD models) that Aβ42 levels are at the limit of detection. LDH and TUNEL assays indicated that inactive STAT3 mutant significantly blocked BDNF-deprivation-induced neuronal cell death, so were the TUNEL and Tau N368 co-staining activities (Figures 3G–3I). Hence, these data support the hypothesis that STAT3 phosphorylation and activation upon BDNF deprivation is essential for C/EBPβ upregulation and the subsequent adverse events.

To further assess the role of C/EBPβ downstream targets in these events, we infected primary neuronal cultures with lentivirus expressing shRNA against C/EBPβ to selectively knock down this transcription factor, followed by anti-BDNF IgG treatment. Immunoblotting revealed that BDNF deprivation-induced C/EBPβ upregulation was prevented by this treatment. Accordingly, the elevated expression of δ-secretase expression, APP

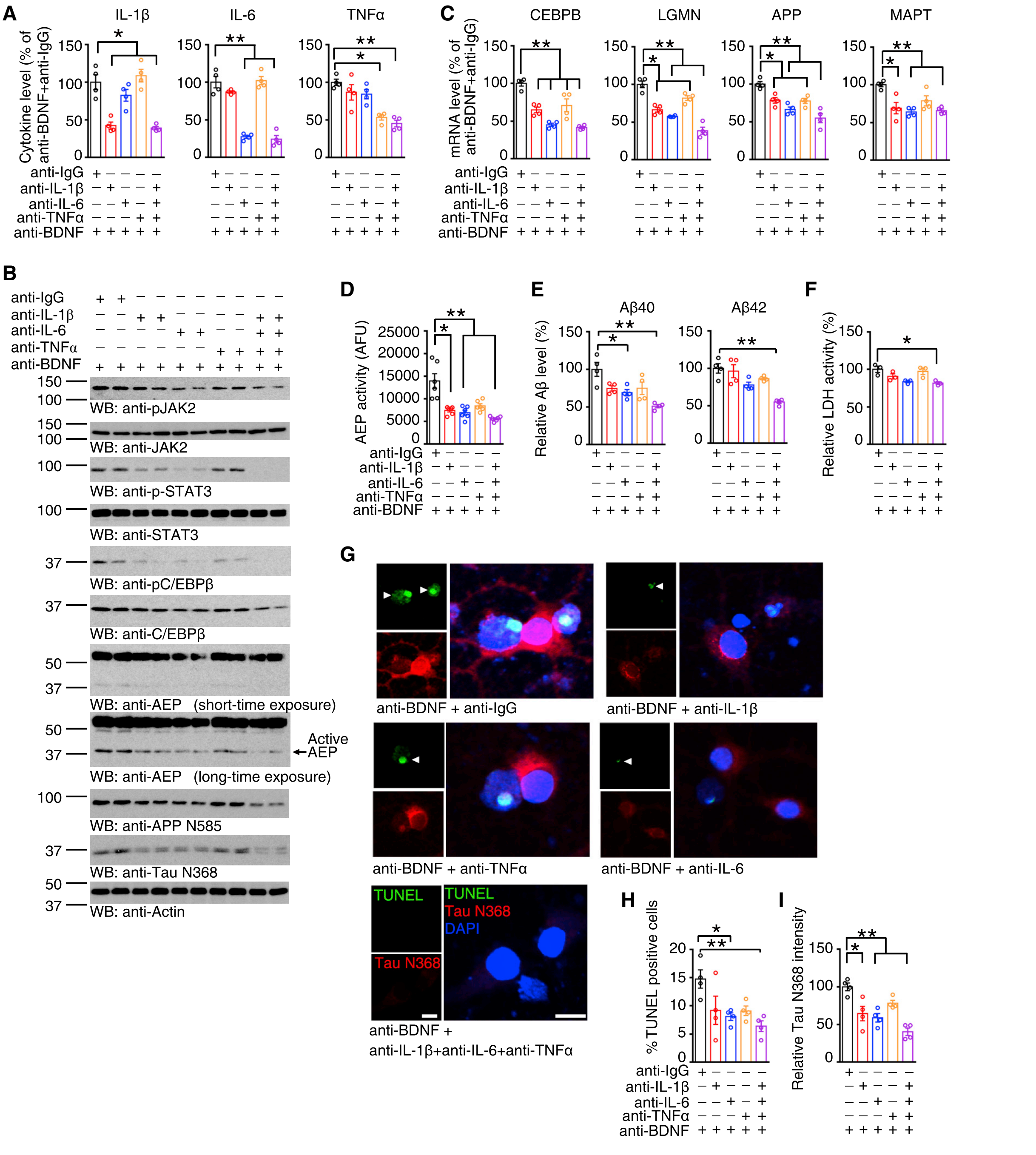


**Figure 2. Neutralizing Cytokines Inhibits STAT3 Activation and C/EBPβ Expression Triggered by BDNF Deprivation**

(A) ELISA of neuronal medium. Data represent mean ± SEM of 4 samples per group from 3 independent experiments (*p < 0.05, **p < 0.01, one-way ANOVA and Bonferroni's multiple comparison test).

(B) Immunoblotting was conducted from BDNF-deprived neurons pretreated with cytokine antibodies. Data are representative of 3 independent experiments.

(C) qRT-PCR analysis. Data represent mean ± SEM of 4 independent experiments (*p < 0.05, **p < 0.01, one-way ANOVA and Bonferroni's multiple comparison test).

(D) δ-Secretase enzymatic activity assay. Data represent mean ± SEM (n = 6, *p < 0.05, one-way ANOVA and Bonferroni's multiple comparison test).

(E) Antibody-mediated blockade of cytokines significantly represses Aβ production induced by BDNF deprivation. Quantification of Aβ levels by ELISA represents mean ± SEM (n = 4, *p < 0.05, **p < 0.01, one-way ANOVA and Bonferroni's multiple comparison test).

(F) LDH assay. Data shown as mean ± SEM of three independent experiments (*p < 0.05, one-way ANOVA and Bonferroni's multiple comparison test).

(G–I) TUNEL and IF double-staining (G). Scale bar, 10 μm. Quantifications of TUNEL positive ecells (H) and Tau N368 level (I) represent mean ± SEM (n = 4, *p < 0.05, **p < 0.05, one-way ANOVA and Bonferroni's multiple comparison test).

See also Figure S2.

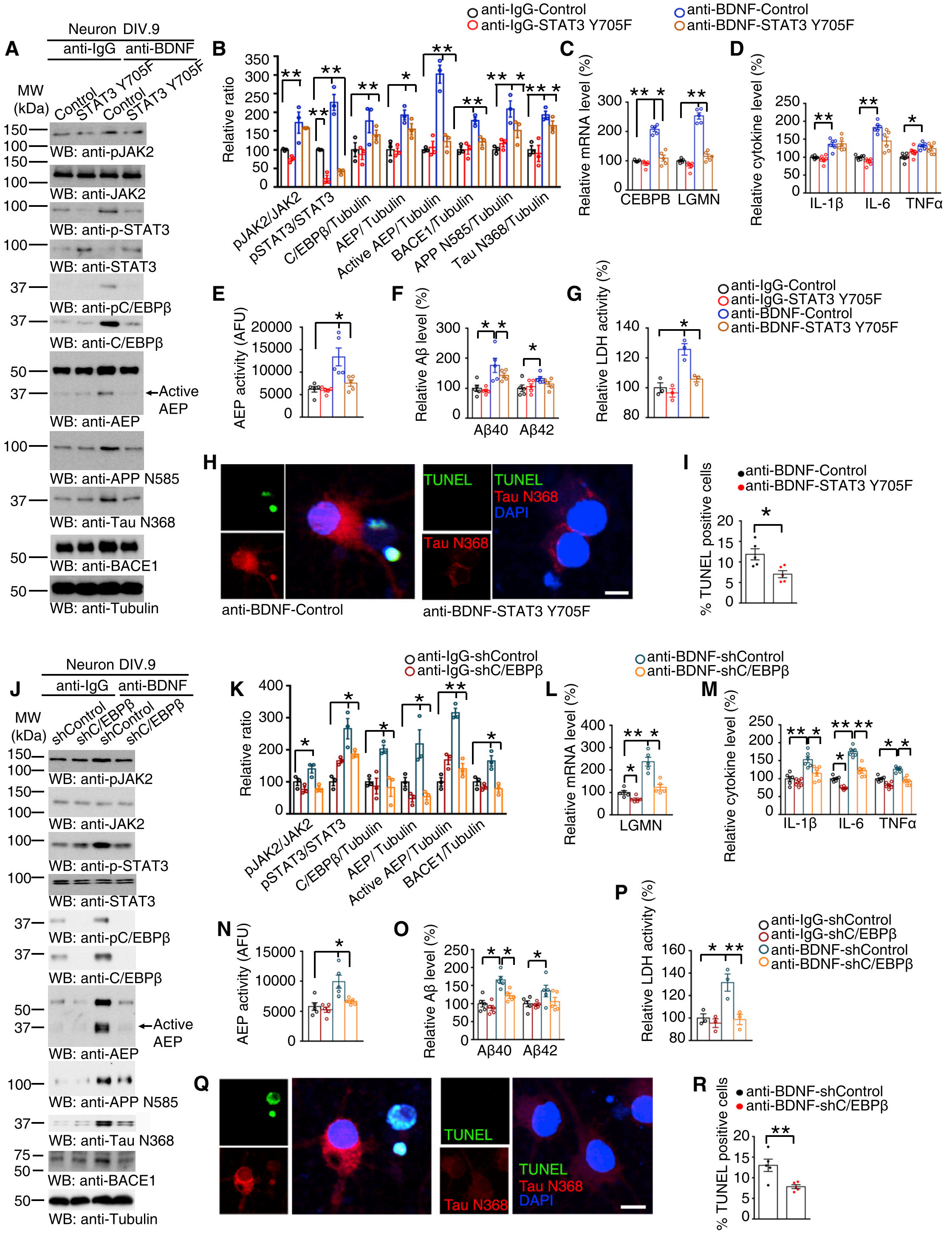


**Figure 3. Blockade of STAT3 or Knockdown of C/EBPβ Rescues BDNF-Deprivation-Induced δ-Secretase Upregulation in Primary Neuron**

(A) STAT3 Y705F reverses the upregulations of C/EBPβ and δ-secretase induced by BDNF deprivation. Western blot data are representative of 3 independent experiments.

(B) Quantification of western blotting (n = 3, $^*p < 0.05$, $^{**}p < 0.01$, one-way ANOVA and Bonferroni's multiple comparison test).

(C) qRT-PCR analysis. Data represent mean ± SEM of 5 independent experiments ($^*p < 0.05$, $^{**}p < 0.01$, one-way ANOVA and Bonferroni's multiple comparison test).

(D) Relative cytokine levels measured by ELISA of cell lysates. Data represent mean ± SEM (n = 6, $^*p < 0.05$, $^{**}p < 0.01$, one-way ANOVA and Bonferroni's multiple comparison test).

(E) δ-Secretase activity assay. Data represent mean ± SEM (n = 5, $^*p < 0.05$, one-way ANOVA and Bonferroni's multiple comparison test).

*(legend continued on next page)*

N585 and Tau N368 proteolytic cleavage, as well as LGMN mRNA and activity were reduced (Figures 3J–3L and 3N). Fitting with previous reports that C/EBPβ acts as the crucial transcription factor for various inflammatory cytokines (Poli, 1998), knockdown of C/EBPβ also reduced their expressions (Figure 3M). Again, Aβ40, but not Aβ42, production triggered by anti-BDNF was selectively antagonized by depletion of C/EBPβ (Figure 3O). BDNF depletion-triggered neuronal cell death was significantly blunted by C/EBPβ knockdown (Figures 3P–3R). Moreover, knockout of AEP prevented the cleavage of Tau and APP and cell death induced by BDNF deprivation (Figures S2D–S2G). These findings suggest that C/EBPβ escalation upon BDNF deprivation is required for downstream LGMN expression and the resultant adverse effects.

## JAK2/STAT3 Activation and C/EBPβ Upregulation in Human AD Patient Brains

To investigate whether BDNF-deprivation-elicited events also occur in the human AD brain, we performed immunoblotting that demonstrated that phosphorylation of JAK2 and STAT3 and the downstream target C/EBPβ were augmented in human AD brains as compared to the age-matched healthy controls. δ-Secretase levels corresponded with C/EBPβ levels, and δ-secretase was selectively truncated and active in AD brains. Moreover, the downstream APP and Tau cleavage products were seen in AD but not control brains (Figures 4A and 4B). BDNF was significantly reduced in AD patient brains as compared to healthy controls (Figure 4C), and inflammatory cytokines were also increased in AD brains (Figure 4D). Co-labeling with BDNF also showed that the levels of p-STAT3, C/EBPβ, δ-secretase, and Tau N368 were inversely increased in AD brains (Figures 4E–4H). To further assess the immediate downstream regulation between C/EBPβ and δ-secretase, we found that both of them were highly elevated in human AD brains and so were δ-secretase and its truncated Tau N368 (Figures 4I and 4J). Quantitative analysis of the fluorescent intensity demonstrated a significant correlation between levels of BDNF and that of p-STAT3, C/EBPβ, and δ-secretase (Figures S3A–S3D). Finally, BDNF reduction and increases in p-STAT3 and δ-secretase were also correlated with an increase in Iba1 in human AD brains (Figures S3E–S3G). These data strongly support that BDNF deficiency in human AD brains elicits p-JAK2/STAT3, C/EBPβ, and δ-secretase upregulation and activation, triggering neuroinflammation.

## BDNF Knockout Activates the JAK2/STAT3 Pathway and Elicits an Impairment in Synaptic Plasticity and Cognitive Deficits

To test whether BDNF deficiency *in vivo* also initiates the similar biological cascades as that seen in primary cultures, $BDNF^{f/f}$ mice were injected either with AAV-GFP or AAV-Cre-GFP in the hippocampus to generate control and BDNF knockout, respectively. To mimic deficient BDNF/TrkB activities, which start from late adulthood (Lommatzsch et al., 2005; Ziegenhorn et al., 2007), $BDNF^{f/f}$ mice were injected at 13.5 months of age, which is equivalent to around 50 years in human (Dutta and Sengupta, 2016). Dual labeling and BDNF ELISA confirmed that transduced (GFP+) hippocampal neurons were massively void of BDNF (Figures S4B–S4G). Because BDNF is essential for neuronal survival, BDNF knockout subsequently triggered caspase-3 activation. Moreover, C/EBPβ levels were also elevated (Figure S4D). As observed by anti-BDNF treatment or BDNF depletion in primary neurons, BDNF knockout induced JAK2/STAT3 activation, and the resultant C/EBPβ and δ-secretase upregulation and activation, ultimately leading to both APP N585 and Tau N368 cleavage (Figures 5A and 5B; Figure S4A). As expected, both CEBPB and LGMN mRNA levels were elevated following BDNF ablation (Figure 5C). Quantitation of fluorescent signal intensities for BDNF reduction, C/EBPβ, active-caspase-3, and Tau N368 escalation confirmed these results (Figure S4F). δ-secretase enzymatic activity was elevated, and pro-inflammatory cytokines were increased (Figures 5D and 5E). BDNF knockout also augmented levels of mouse Aβ (Figure 5F). Levels of Iba1 were also elevated following BDNF knockout, as well as both anti-Aβ and p-Tau (AT8), which detected endogenous mouse Aβ and pTau (Figure 5G). Golgi staining showed that dendritic spines were significantly reduced following BDNF removal (Figure 5H). Moreover, electronic microscopic (EM) analysis demonstrated a marked reduction in synapses in the BDNF-depleted hippocampus (Figure 5I). Long-term potentiation (LTP), a form of plasticity, underlies the cellular mechanism of learning and memory (Bliss and Collingridge, 1993). Electrophysiological measurements showed that LTP was substantially reduced in the BDNF-deleted hippocampus (Figure 5J).

(F) Quantification of Aβ levels by ELISA represents mean ± SEM (n = 5,*$p < 0.05$, **$p < 0.01$, one-way ANOVA and Bonferroni's multiple comparison test).
(G) LDH assay. Data shown as mean ± SEM of 3 independent experiments (*$p < 0.05$, one-way ANOVA and Bonferroni's multiple comparison test).
(H and I) TUNEL and IF double-staining (H). Scale bar, 10 μm. Quantification (I) represents mean ± SEM (n = 5, *$p < 0.05$, unpaired t test with Welch's correction).
(J) C/EBPβ knockdown decreases C/EBPβ expression. Data are representative of 3 independent experiments.
(K) Quantification of western blotting (n = 3, *$p < 0.05$, **$p < 0.01$, one-way ANOVA and Bonferroni's multiple comparison test).
(L) qRT-PCR analysis. Data represent mean ± SEM of 5 independent experiments (*$p < 0.05$, **$p < 0.01$, one-way ANOVA and Bonferroni's multiple comparison test).
(M) Cytokine levels as measured by ELISA. Data represent mean ± SEM of 6 samples per group (*$p < 0.05$, **$p < 0.01$, one-way ANOVA and Bonferroni's multiple comparison test).
(N) BDNF-deprivation-induced active δ-secretase is mitigated by C/EBPβ knockdown. Data represent mean ± SEM (n = 5, *$p < 0.05$, one-way ANOVA and Bonferroni's multiple comparison test).
(O) Quantification of Aβ levels by ELISA represents mean ± SEM (n = 5,*$p < 0.05$, **$p < 0.01$, one-way ANOVA and Bonferroni's multiple comparison test).
(P) LDH assay. Data shown as mean ± SEM of 3 independent experiments (*$p < 0.05$, one-way ANOVA and Bonferroni's multiple comparison test).
(Q and R) TUNEL and IF staining (Q). Scale bar, 10 μm. Quantification (R) represents mean ± SEM (n = 5, **$p < 0.01$, unpaired t test with Welch's correction).
See also Figure S2.

OPEN
ACCESS
CellPress

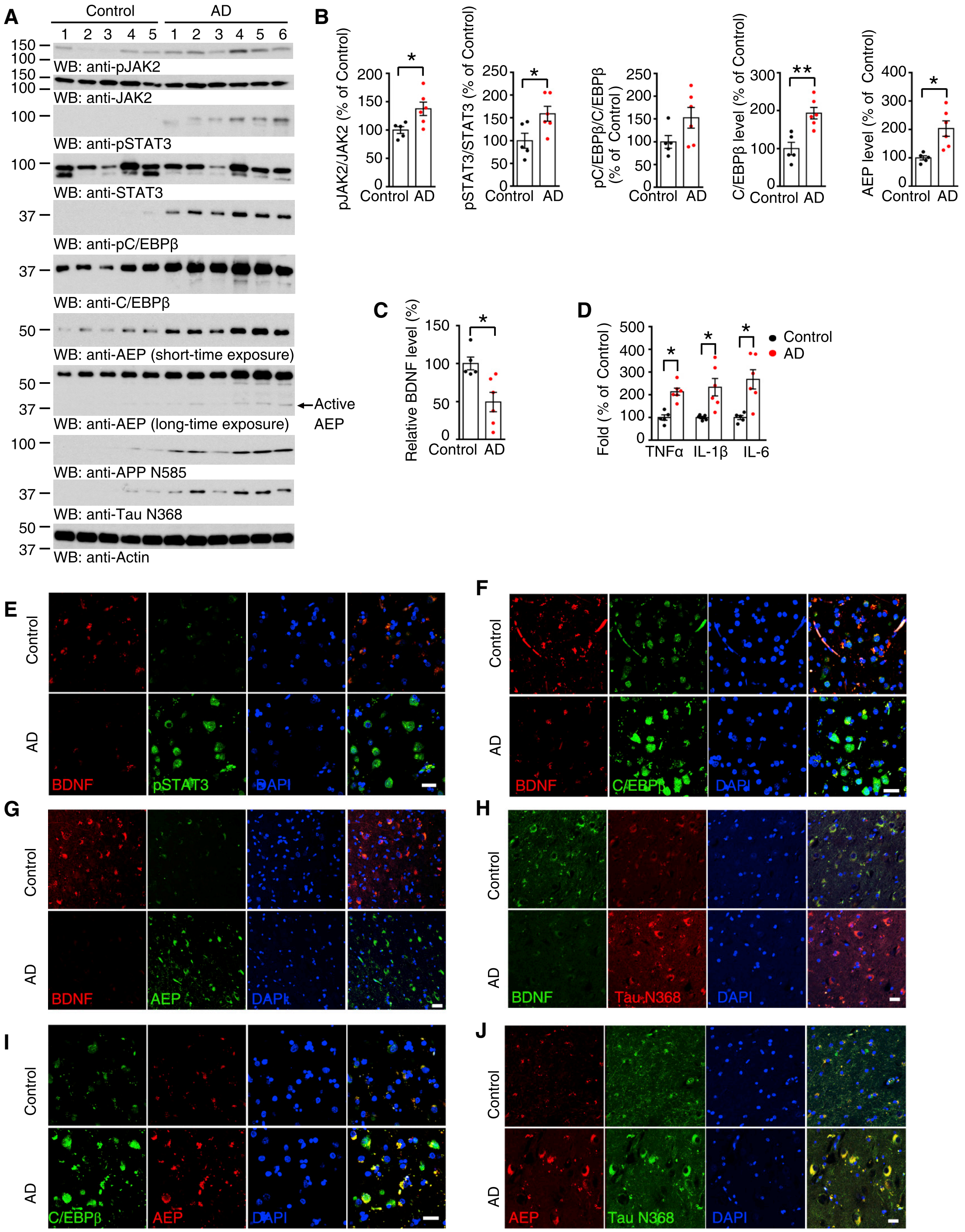


**Figure 4. JAK2/STAT3 Activation and C/EBPβ Upregulation in Human AD Brains**

(A and B) Western blot detection of JAK2/STAT3 pathway and C/EBPβ/AEP levels in human brain samples from subjects with AD and age-matched controls (A) and the quantifications (B). Data represent mean ± SEM (n = 5 for control; n = 6 for AD; *p < 0.05, **p < 0.01, unpaired t test with Welch's correction).

(C and D) ELISA detection of BDNF (C) and cytokine (D) levels in human brain samples.

(E–J) IF staining of BDNF/pSTAT3 (E), BDNF/C/EBPβ (F), BDNF/AEP (G), BDNF/Tau N368 (H), C/EBPβ/AEP (I), and AEP/Tau N368 (J) in human hippocampus samples. Scale bars, 20 μm. IF data are representative of 5 or 6 independent cases.

See also Figure S3.

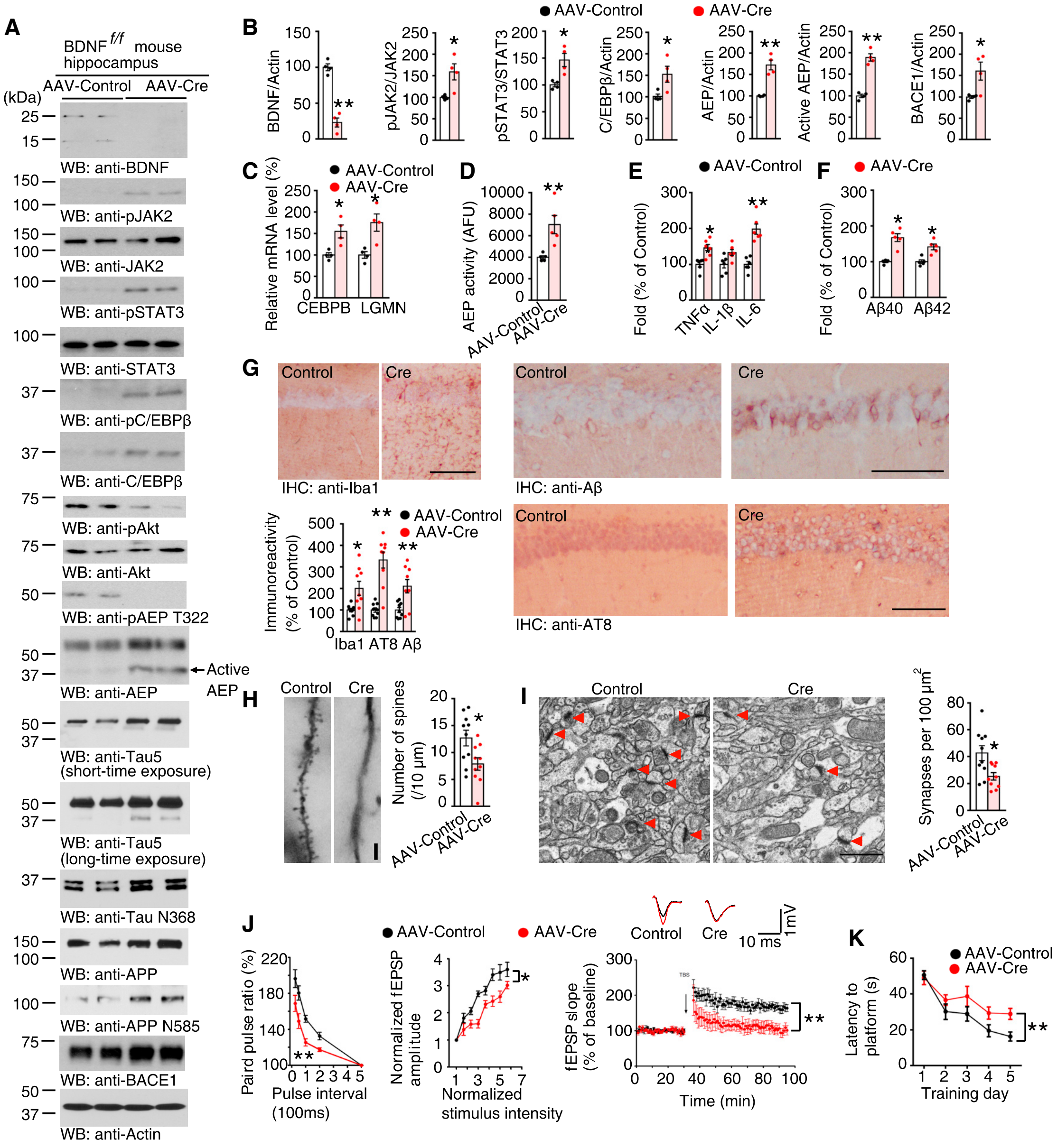


**Figure 5. Knockout of BDNF Activates JAK2/STAT3 Pathway and Elicits Synaptic Plasticity Impairment and Cognitive Deficits**

(A) BDNF knockout activates JAK2/STAT3 and increases the expression of C/EBPβ and δ-secretase. Immunoblotting was conducted from 16-month-old BDNF$^{f/f}$ mice hippocampus injected with AAV-Cre or AAV-GFP. Data are representative of four mice.

(B) Quantification of western blotting (n = 4, *p < 0.05, **p < 0.01, unpaired t test with Welch's correction).

(C) qRT-PCR analysis. Data represent mean ± SEM of 4 mice (**p < 0.01, unpaired t test with Welch's correction).

(D) δ-secretase enzymatic activities. Data represent mean ± SEM (n = 5, **p < 0.01, unpaired t test with Welch's correction).

(E) BDNF knockout stimulates inflammatory cytokine production. Data represent mean ± SEM (n = 6, *p < 0.05, **p < 0.01, unpaired t test with Welch's correction).

(F) Quantification of Aβ levels by ELISA represents mean ± SEM (n = 5,*p < 0.05, **p < 0.01, unpaired t test with Welch's correction).

(G) IHC. Scale bar, 50 μm. Data shown as mean ± SEM of 9 sections from 3 mice in each group (*p < 0.05, unpaired t test with Welch's correction).

(H) Golgi staining was conducted on brain sections from apical dendritic layer of the CA1 region. Scale bar, 5 μm. Data represent mean ± SEM of 10 sections from 3 mice in each group. (*p < 0.05, **p < 0.01, unpaired t test with Welch's correction).

(I) Electronic microscopic (EM) analysis. Scale bar, 1 μm. Data represent mean ± SEM of 10 sections from 3 mice in each group. (*p < 0.05, unpaired t test with Welch's correction).

(J) Electrophysiology analysis (mean ± SEM; n = 6 in each group; *p < 0.05, **p < 0.01, two-way ANOVA and Bonferroni's post hoc test). The ratio of paired pulses in different groups (left). Input-output curve (middle). LTP (right). Shown traces were representative field excitatory post-synaptic potentials (fEPSPs) recorded before (black) and 60 min after (red) Tris-buffered saline (TBS).

(K) Morris Water Maze analysis (mean ± SEM; n = 8-9 mice per group; **p < 0.01, two-way ANOVA and Bonferroni's post hoc test).

See also Figures S4 and S5.

Consistent with these findings, depletion of BDNF strongly reduced learning and memory in a Morris Water Maze (MWM) behavioral test of BDNF-ablated mice without affecting its motor functions (Figure 5K; Figures S4H–S4J). Fear-conditioning testing also confirmed that deletion of BDNF impaired memory function (Figure S4K). The corresponding observations were made in transgenic homozygous TrkB-floxed mice (TrkB$^{f/f}$) injected with AAV-Cre (Figure S5). Thus, knockout of BDNF or TrkB in the hippocampus results in JAK2/STAT3, C/EBPβ, and δ-secretase upregulation and activation, resulting in increased neuroinflammation and neuronal cell death, leading to cognitive impairments.

### STAT3 Y705F Prevents BDNF-Depletion-Mediated Pathology

To examine whether BDNF-knockout-mediated C/EBPβ upregulation and downstream events are mediated by activation of the JAK2/STAT3 pathway, we co-injected the hippocampus of BDNF$^{f/f}$ mice with a mix of vectors expressing either Cre or STAT3 Y705F mutant. IF staining showed that expression of the STAT3 Y705F mutant completely blocked BDNF-knockdown-mediated phosphorylation of STAT3 (Figures S6A and S6B). Importantly, the activation of caspase 3 induced by BDNF knockout was strongly blocked in the presence of STAT3 mutant expression (Figures S6A and S6B). Moreover, BDNF-deletion-triggered C/EBPβ was blocked, leading to a reduction of δ-secretase and the resultant decrease in APP N585 and Tau N368 cleavage (Figures 6A and 6B). Again, both C/EBPβ and LGMN mRNA were reduced in the presence of the inactive STAT3 mutant, as was δ-secretase enzymatic activity (Figures 6C and 6D). As expected, the production of pro-inflammatory cytokines was reduced by STAT3 Y705F expression (Figure 6E), and Aβ40 and Aβ42 levels in the hippocampal lysates were both reduced (Figure 6F). Immunohistochemistry (IHC) analysis showed an increase in hippocampal Iba1 as well as Aβ, and pTau in BDNF knockout brains was robustly repressed by STAT3 Y705F (Figure 6G). On the contrary, dendritic spine density and synapses were both greatly enhanced by STAT3 mutant expression, as demonstrated by Golgi staining and EM, respectively (Figures 6H and 6I). Consistent with these findings, an electrophysiological analysis indicated that STAT3 mutant expression resulted in increased LTP as compared to control (Figure 6J). In agreement with these findings, the MWM and fear-conditioning assays showed that the STAT3 Y705F mutant rescued the learning and memory deficits triggered by BDNF depletion (Figures 6K and 6L; Figures S6C–S6E). In conclusion, STAT3 Y705 phosphorylation is a crucial mediator of the detrimental effects that occurred following hippocampal BDNF knockout.

### Depletion of C/EBPβ or Inhibition of δ-Secretase Restores BDNF-Knockout-Triggered Murine Aβ and Tau Alterations

To delineate the role of C/EBPβ in BDNF-knockout-initiated pathological events, we co-injected viruses expressing a C/EBPβ shRNA and Cre recombinase into the hippocampus of BDNF$^{f/f}$ mice. IF staining and immunoblotting confirmed a substantial reduction of C/EBPβ expression. C/EBPβ knockdown repressed BDNF-depletion-induced caspase 3 activity (Figures 7A and 7B). As a result of C/EBPβ knockdown, δ-secretase was reduced, leading to inhibition of both APP N585 and Tau N368 cleavage (Figures 7C and 7D). RT-PCR and a δ-secretase enzymatic assay confirmed these observations (Figures 7E and 7F). Elimination of C/EBPβ significantly reduced BDNF-depletion-elevated IL-6, which was expected as IL-6 is the immediate downstream target of C/EBPβ (Figure 7G). Furthermore, both Aβ40 and 42 were clearly reduced upon C/EBPβ knockdown (Figure 7H). IHC analysis mirrored these findings and p-Tau was reduced (Figure 7I). Both dendritic spine density and synapses were greatly augmented after C/EBPβ was knocked down in the BDNF-depleted hippocampus (Figures 7J and JK). Electrophysiology analysis demonstrated that deletion of C/EBPβ significantly elevated LTP (Figure 7L; Figure S5F). Behavioral assays validated that knockdown of C/EBPβ alleviated BDNF-depletion-triggered learning and memory deficits (Figures 7M and 7N; Figures S5G–S5I).

As an immediate downstream target gene of C/EBPβ, δ-secretase activity is tightly regulated by C/EBPβ in AD brains (Wang et al., 2018a). To explore whether δ-secretase is responsible for C/EBPβ-mediated murine Aβ and Tau alterations in the BDNF-depleted hippocampus, we treated BDNF$^{f/f}$ mice with a dominant-negative δ-secretase C189S mutant together with AAV-Cre. IF staining confirmed that δ-secretase was highly expressed in the transduced hippocampus, and BDNF-knockout-induced active caspase-3 was repressed by the expression of C189S mutant (Figures S7A and S7B). Immunoblotting indicated that C189S overexpression blocked δ-secretase activation, resulting in suppression of Tau N368 and APP N585 cleavage. Noticeably, JAK2/STAT3 phosphorylation and C/EBPβ expression and activation were reduced by expression of the δ-secretase mutant (Figures S7C and S7D), suggesting that δ-secretase also feeds back and mediates upstream transcription factor activation. This was validated by RT-PCR analysis of C/EBPβ mRNA (Figure S7E). δ-secretase enzymatic activity was suppressed by its inactive mutant, as was the production of inflammatory cytokines and Aβ (Figures S7F–S7H). IHC analysis showed that inhibiting δ-secretase in the BDNF-deleted hippocampus reduced Iba1, Aβ, and p-Tau (Figure S7I). Similar to depletion of C/EBPβ, inhibition of δ-secretase activity by the C189S mutant also restored dendritic spines and synapse density, resulting in alleviation of LTP impairments and cognitive deficits induced by BDNF depletion in the hippocampus (Figures S7J–S7Q). Therefore, blockade of C/EBPβ or its downstream effector δ-secretase mitigates the murine Aβ and Tau alterations and cognitive defects elicited by BDNF knockout.

## DISCUSSION

BDNF/TrkB neurotrophic activity plays a crucial role in synaptic plasticity and neuronal survival (Minichiello, 2009). In previous 3xTg/BDNF +/− animal models, BDNF gene dose was reduced in half. However, the neurotrophic signalings were relatively intact. To interrogate whether BDNF may be mechanistically involved in the pathogenesis of AD, Castello et al. (2012) showed that 3xTg/BDNF +/− mice display comparable Aβ and Tau pathologies as 3xTg/BDNF +/+ mice. Hence, they suggest that chronic reduction of BDNF does not exacerbate the

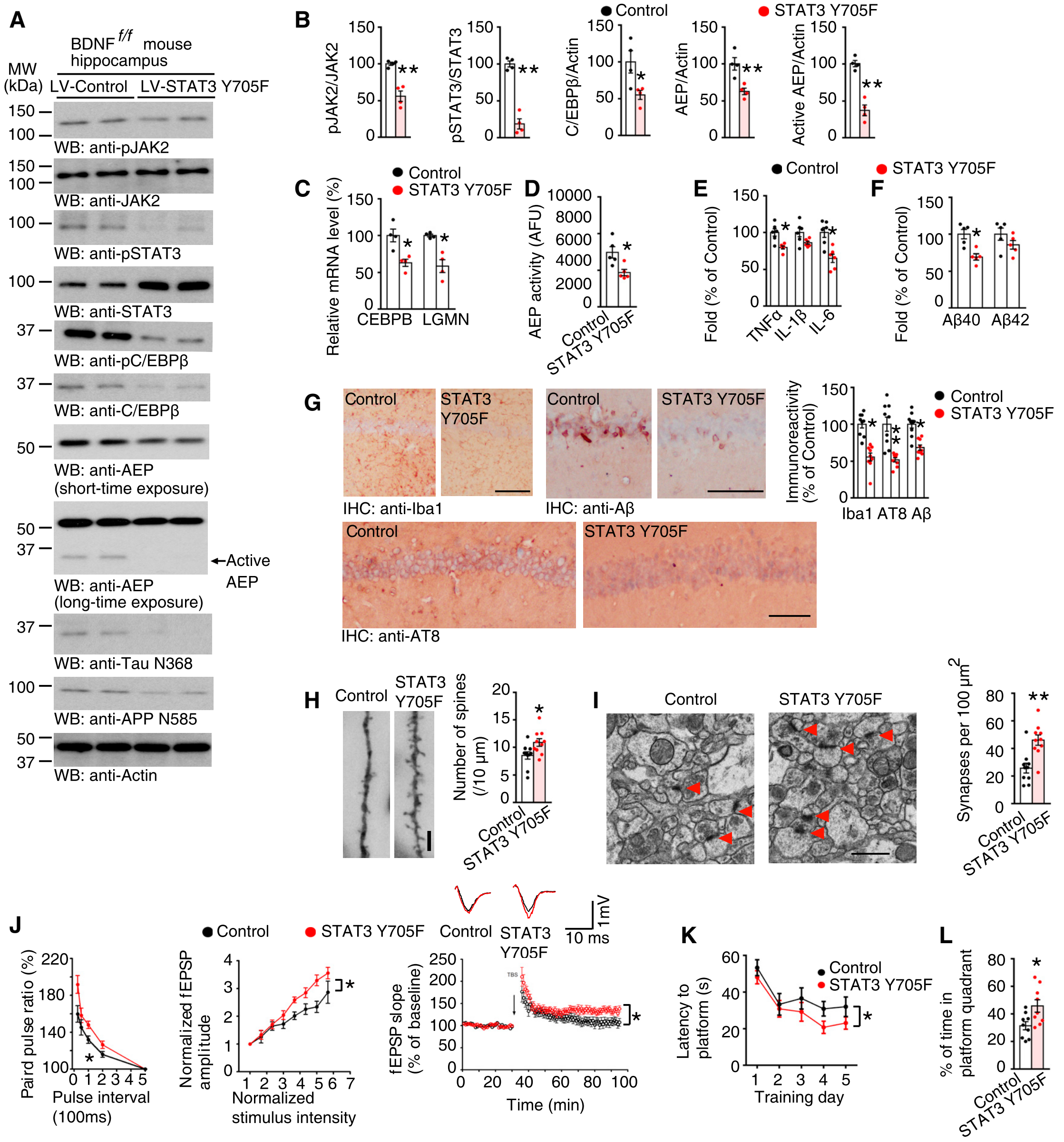


**Figure 6. Expression of STAT3 Y705F Blocks BDNF-Depletion-Induced Murine Aβ and Tau Alterations**

(A) Immunoblotting was conducted from 16-month-old BDNF flox/flox mice hippocampus co-injected with AAV-Cre and LV-Control or AAV-Cre and LV- STAT3 Y705F. Data were representatives of three independent experiments.

(B) Quantification of western blotting (n = 4, *p < 0.05, **p < 0.01, unpaired t test with Welch's correction). (C) qRT-PCR analysis. Data represent mean ± SEM (n = 4, **p < 0.01, unpaired t test with Welch's correction).

(D) δ-Secretase activity assay. Data represent mean ± SEM (n = 5, *p < 0.05, unpaired t test with Welch's correction).

(E) Relative cytokine levels measured by ELISA of hippocampus lysates. Data represented mean ± SEM (n = 6, *p < 0.05, **p < 0.01, unpaired t test with Welch's correction).

(F) Quantification of Aβ levels by ELISA represents mean ± SEM (n = 5, *p < 0.05, **p < 0.01, unpaired t test with Welch's correction).

(G) IHC. Scale bars, 50 μm. Data shown as mean ± SEM (n = 9 sections, *p < 0.05, unpaired t test with Welch's correction).

(H) Golgi staining of CA1. Scale bar, 5 μm. Data represent mean ± SEM (n = 9–10 sections, *p < 0.05, unpaired t test with Welch's correction).

(I) EM analysis. Scale bar, 1 μm. Data represent mean ± SEM (n = 10 sections, *p < 0.05, unpaired t test with Welch's correction).

(J) Electrophysiology analysis (mean ± SEM; n = 6 in each group; *p < 0.05, two-way ANOVA and Bonferroni's post hoc test). The ratio of paired pulses in different groups (left). Input-output curves (middle). LTP of fEPSPs (right). Shown traces are representative fEPSPs recorded before (black) and 60 min after (red) TBS.

(K and L) Morris Water Maze analysis (mean ± SEM; n = 9 mice per group; *p < 0.05, two-way ANOVA and Bonferroni's post hoc test for K and unpaired t test with Welch's correction for L).

See also Figure S6.

OPEN ACCESS
CellPress

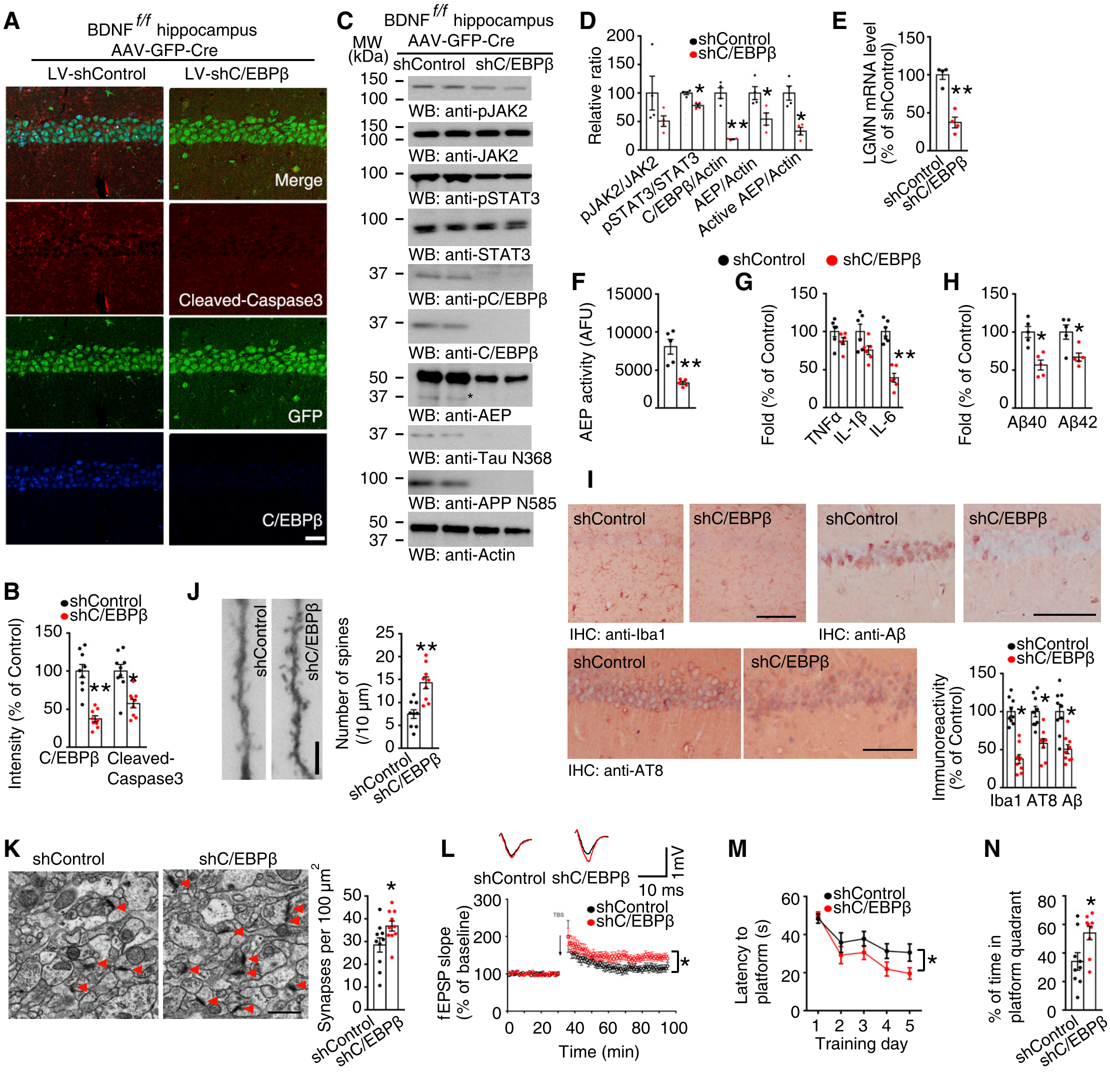

**Figure 7. Knockdown of C/EBPβ Restores BDNF-Knockout-Mediated Neuronal Apoptosis and Murine Aβ and Tau Alterations**

(A) IF staining of cleaved-caspase 3 and C/EBPβ. Scale bar, 60 μm.

(B) Quantification of fluorescent intensity represents mean ± SEM (n = 9, *p < 0.05, **p < 0.01, unpaired t test with Welch's correction).

(C) Immunoblotting was conducted from 16-month-old $BDNF^{f/f}$ mice hippocampus co-injected with AAV-Cre and LV-shControl or AAV-Cre and LV- shC/EBPβ. Data are representatives of three independent experiments. *Active AEP.

(D) Quantification of western blotting (n = 4, *p < 0.05, unpaired t test with Welch's correction).

(E) qRT-PCR analysis. Data represent mean ± SEM (n = 4, **p < 0.01, unpaired t test with Welch's correction).

(F) δ-Secretase activity assay. Data represent mean ± SEM (n = 5, *p < 0.05, unpaired t test with Welch's correction).

(G) Relative cytokine levels. Data represent mean ± SEM (n = 6, *p < 0.05, **p < 0.01, unpaired t test with Welch's correction).

(H) Quantification of Aβ levels represents mean ± SEM (n = 5, *p < 0.05, **p < 0.01, unpaired t test with Welch's correction).

(I) IHC in CA1. Scale bar, 50 μm. Data shown as mean ± SEM (n = 9 sections, *p < 0.05, unpaired t test with Welch's correction).

(J) Golgi staining. Scale bar, 5 μm. Data represent mean ± SEM (n = 9 sections, *p < 0.05, unpaired t test with Welch's correction).

(K) EM analysis. Scale bar, 1 μm. Data represent mean ± SEM of 10 sections from 3 mice in each group. (n = 10 sections, *p < 0.05, unpaired t test with Welch's correction).

(L) LTP of fEPSPs (mean ± SEM; n = 6 in each group; **p < 0.01, two-way ANOVA and Bonferroni's post hoc test). Shown traces are representative fEPSPs recorded before (black) and 60 min after (red) TBS.

(M and N) Morris Water Maze analysis during the training period (M) and probe trial (N) (mean ± SEM; n = 9 mice per group; *p < 0.05, one-way ANOVA and Bonferroni's multiple comparison test).

See also Figures S6 and S7.

development of Aβ and Tau pathology and instead suggest the reduced BDNF levels found in AD patients are a consequence of these pathologies (Castello et al., 2012). On the other hand, although p-TrkB signaling is reduced in 5xFAD/TrkB +/− mice, there was no difference between 5XFAD/TrkB +/− and 5XFAD/TrkB +/+ control mice in cerebral plaque loads, Aβ concentrations, including total Aβ42 and soluble oligomers, and β-amyloidogenic processing of amyloid precursor protein. The authors found reduced TrkB does not affect β-amyloidosis but exacerbates the manifestation of hippocampal mnemonic and signaling dysfunctions in early AD (Devi and Ohno, 2015).

In our current study, we are not addressing human Aβ or Tau pathology in AD mouse models but instead non-pathological elevations of murine Aβ and Tau in wild-type mice elicited by BDNF/TrkB deprivation, which might be the main explanation of this discrepancy between our study and previous reports. Indeed, mouse Aβ might display different aggregates from human Aβ in senile plaques morphology (Xu et al., 2015). Clearly, our study provides insight into BDNF/TrkB pathway and its potential contribution in AD-like pathologies.

Recent reports indicate that STAT3 acts as a critical transcription factor for C/EBPβ (Zhang et al., 2011), a key regulator for δ-secretase (Wang et al., 2018a). Inactivation of STAT3 also repressed cytokine production (Figure 6), fitting with a previous report that STAT3 regulates cytokine expression (Agrawal et al., 2011). In addition, this result is consistent with the finding that C/EBPβ, in concert with nuclear factor κB (NF-κB), mediates inflammatory cytokines expression (Poli, 1998). For instance, C/EBPβ is a major IL-6 transcription factor (Isshiki et al., 1990; Wedel and Ziegler-Heitbrock, 1995). Mounting evidence implicates C/EBPβ in neuro-inflammation (Ejarque-Ortiz et al., 2007), and this protein is upregulated in human AD brains (Strohmeyer et al., 2014; Wang et al., 2018a).

The expression of inflammatory cytokines is elevated in the CNS in diverse neurodegenerative disorders, including AD. Our findings support that impaired BDNF/TrkB neurotrophic activity contributes to this event. On the other hand, IL-1β also impairs BDNF-induced signal transduction (Tong et al., 2008). Neuroinflammation activates the JAK2/STAT3 pathway in the brain (O'Sullivan et al., 2007; Wang et al., 2014). Moreover, aging, one of the most common risk factors for AD, also correlates with neuronal inactivation of STAT3; p-STAT3 immunoreactivity in hippocampal neurons of young subjects is substantially higher than that of older cognitively normal subjects in both humans and rodents (Chiba et al., 2009).

Interestingly, neurons containing NFTs do not contain BDNF immuno-reactivity, whereas neurons strongly immunoreactive for BDNF are devoid of tangles (Murer et al., 1999), suggesting that BDNF may have a protective role against AD pathogenesis. Several immediate early (IE) genes act as targets for BDNF/TrkB signaling for neuronal functions, and C/EBPβ is recruited to the target promoter upon BDNF treatment (Calella et al., 2007). Moreover, C/EBPs and Trks are required for cortical dendrite differentiation, and Trks regulate dendritic differentiation by a C/EBP-dependent mechanism. Thus, BDNF induction of genes important for neuronal functions depends on transcription factors, including C/EBPβ upregulation during neuronal development (Calella et al., 2007). Furthermore, BDNF mediates the maintenance of memory consolidation. A hippocampal BDNF-positive auto-regulatory feedback loop mediated by C/EBPβ is necessary for memory consolidation. On the other hand, C/EBPβ controls expression of *bdnf* exon IV transcripts, thus mediating memory consolidation (Bambah-Mukku et al., 2014). Thus, C/EBPβ plays a critical role in mediating BDNF function, and these two proteins mutually regulate each other in the CNS. Clearly, these beneficial immediate early responses between BDNF and C/EBPβ sharply contrast the detrimental effects triggered by sustained BDNF deprivation by upregulating C/EBPβ. Conceivably, the constructive or destructive roles of C/EBPβ in learning and memory might be dictated by the duration, availability, and abundance of BDNF. Together, our findings support that BDNF/TrkB scarcity in AD elicits C/EBPβ upregulation by the p-JAK2/p-STAT3 pathway activated by inflammatory cytokines, and active C/EBPβ subsequently escalates δ-secretase expression that mediates APP and Tau pathological processing, culminating in massive neuronal cell loss. Conceivably, JAK2 or AEP inhibitors may block BDNF/TrkB neurotrophic-signaling-reduction-elicited Aβ and Tau alterations.

The current study mainly focuses on dissecting BDNF/TrkB deprivation and neurotrophic pathway potential contribution to endogenous murine Aβ production and Tau aggregation by the C/EBPβ/AEP pathway. In human AD patients, the pathological alteration is much more complicated. In addition to neurotrophic signaling reduction, they are simultaneously suffering numerous abnormal stresses, including hypercholesterol, diabetes, and chronic inflammation elicited either by traumatic injury or oxidative stress. Hence, our model sheds light on the biological implication of BDNF/TrkB in regulating the C/EBPβ/AEP pathway, and its potential involvement in regulating murine Aβ and Tau biology.

## STAR★METHODS

Detailed methods are provided in the online version of this paper and include the following:

- KEY RESOURCES TABLE
- LEAD CONTACT AND MATERIALS AVAILABILITY
- EXPERIMENTAL MODEL AND SUBJECT DETAILS
  - Mice
  - Primary cultured neurons
  - Human tissue samples
- METHOD DETAILS
  - Transfection and infection of the cells
  - BDNF deprivation
  - Cytokines blocked by neutralizing antibodies
  - AEP activity assay
  - Immunoprecipitation and western blot analysis
  - Immunohistochemistry
  - Electron Microscopy
  - Stereotaxic injection in mouse hippocampus
  - Golgi staining
  - Electrophysiology
  - Morris Water maze
  - Contextual fear conditioning
- QUANTIFICATION AND STATISTICAL ANALYSIS

## SUPPLEMENTAL INFORMATION

Supplemental Information can be found online at https://doi.org/10.1016/j.celrep.2019.06.054.

## ACKNOWLEDGMENTS

The authors are indebted to Dr. Arthur English at Emory University for critically proofreading the manuscript. We thank Dr. James O. McNamara for providing $TrkB^{f/f}$ mice; Dr. Colin Watts at College of Life Sciences, University of Dundee, Dundee for the gift of AEP 6E3 antibody; and Dr. Riqiang Yan for BACE1 knockout (KO) brain samples. We thank ADRC at Emory University for human AD patients and healthy control samples. This work was supported by an NIH grant (NIA RF1 AG051538) to K.Y. This work was also partially supported by the China Postdoctoral Science Foundation (2016M590695 and 2017T100558) to Z.-H.W. This study was supported in part by the Rodent Behavioral Core (RBC), which is subsidized by the Emory University School of Medicine and is one of the Emory Integrated Core Facilities. Additional support was provided by the Emory Neuroscience NINDS Core Facilities (P30NS055077). Further support was provided by the Georgia Clinical & Translational Science Alliance of the NIH under award number UL1TR002378. The content is solely the responsibility of the authors and does not necessarily reflect the official views of the NIH.

## AUTHOR CONTRIBUTIONS

K.Y. conceived the project, designed the experiments, analyzed the data, and wrote the manuscript. Z.-H.W. designed and performed most of the experiments. J.X. and X.L. prepared primary neurons and assisted with animal experiments. I.M.S. and F.P.M. provided clones, generated viral genomes, and packaged viral vectors. S.P.Y., F.P.M., S.W., and J.-Z.W. assisted with data analysis and critically read the manuscript.

## DECLARATION OF INTERESTS

The authors declare that they have no conflict of interest.

Received: November 27, 2018
Revised: March 29, 2019
Accepted: June 14, 2019
Published: July 16, 2019

## REFERENCES

Agrawal, S., Gollapudi, S., Su, H., and Gupta, S. (2011). Leptin activates human B cells to secrete TNF-α, IL-6, and IL-10 via JAK2/STAT3 and p38MAPK/ERK1/2 signaling pathway. J. Clin. Immunol. *31*, 472–478.

Akiyama, H., Barger, S., Barnum, S., Bradt, B., Bauer, J., Cole, G.M., Cooper, N.R., Eikelenboom, P., Emmerling, M., Fiebich, B.L., et al. (2000). Inflammation and Alzheimer's disease. Neurobiol. Aging *21*, 383–421.

Ando, S., Kobayashi, S., Waki, H., Kon, K., Fukui, F., Tadenuma, T., Iwamoto, M., Takeda, Y., Izumiyama, N., Watanabe, K., and Nakamura, H. (2002). Animal model of dementia induced by entorhinal synaptic damage and partial restoration of cognitive deficits by BDNF and carnitine. J. Neurosci. Res. *70*, 519–527.

Arancibia, S., Silhol, M., Moulière, F., Meffre, J., Höllinger, I., Maurice, T., and Tapia-Arancibia, L. (2008). Protective effect of BDNF against beta-amyloid induced neurotoxicity in vitro and in vivo in rats. Neurobiol. Dis. *31*, 316–326.

Arnaud, L., Chen, S., Liu, F., Li, B., Khatoon, S., Grundke-Iqbal, I., and Iqbal, K. (2011). Mechanism of inhibition of PP2A activity and abnormal hyperphosphorylation of tau by I2(PP2A)/SET. FEBS Lett. *585*, 2653–2659.

Ballatore, C., Lee, V.M., and Trojanowski, J.Q. (2007). Tau-mediated neurodegeneration in Alzheimer's disease and related disorders. Nat. Rev. Neurosci. *8*, 663–672.

Bambah-Mukku, D., Travaglia, A., Chen, D.Y., Pollonini, G., and Alberini, C.M. (2014). A positive autoregulatory BDNF feedback loop via C/EBPβ mediates hippocampal memory consolidation. J. Neurosci. *34*, 12547–12559.

Berishaj, M., Gao, S.P., Ahmed, S., Leslie, K., Al-Ahmadie, H., Gerald, W.L., Bornmann, W., and Bromberg, J.F. (2007). Stat3 is tyrosine-phosphorylated through the interleukin-6/glycoprotein 130/Janus kinase pathway in breast cancer. Breast Cancer Res. *9*, R32.

Bliss, T.V., and Collingridge, G.L. (1993). A synaptic model of memory: long-term potentiation in the hippocampus. Nature *361*, 31–39.

Calella, A.M., Nerlov, C., Lopez, R.G., Sciarretta, C., von Bohlen und Halbach, O., Bereshchenko, O., and Minichiello, L. (2007). Neurotrophin/Trk receptor signaling mediates C/EBPalpha, -beta and NeuroD recruitment to immediate-early gene promoters in neuronal cells and requires C/EBPs to induce immediate-early gene transcription. Neural Dev. *2*, 4.

Castello, N.A., Green, K.N., and LaFerla, F.M. (2012). Genetic knockdown of brain-derived neurotrophic factor in 3xTg-AD mice does not alter Aβ or tau pathology. PLoS One *7*, e39566.

Chiba, T., Yamada, M., Sasabe, J., Terashita, K., Shimoda, M., Matsuoka, M., and Aiso, S. (2009). Amyloid-beta causes memory impairment by disturbing the JAK2/STAT3 axis in hippocampal neurons. Mol. Psychiatry *14*, 206–222.

Connor, B., Young, D., Yan, Q., Faull, R.L., Synek, B., and Dragunow, M. (1997). Brain-derived neurotrophic factor is reduced in Alzheimer's disease. Brain Res. Mol. Brain Res. *49*, 71–81.

Devi, L., and Ohno, M. (2015). TrkB reduction exacerbates Alzheimer's disease-like signaling aberrations and memory deficits without affecting β-amyloidosis in 5XFAD mice. Transl. Psychiatry *5*, e562.

Dutta, S., and Sengupta, P. (2016). Men and mice: relating their ages. Life Sci. *152*, 244–248.

Ejarque-Ortiz, A., Medina, M.G., Tusell, J.M., Pérez-González, A.P., Serratosa, J., and Saura, J. (2007). Upregulation of CCAAT/enhancer binding protein beta in activated astrocytes and microglia. Glia *55*, 178–188.

Fan, Z., Beresford, P.J., Oh, D.Y., Zhang, D., and Lieberman, J. (2003). Tumor suppressor NM23-H1 is a granzyme A-activated DNase during CTL-mediated apoptosis, and the nucleosome assembly protein SET is its inhibitor. Cell *112*, 659–672.

Ferrer, I., Marín, C., Rey, M.J., Ribalta, T., Goutan, E., Blanco, R., Tolosa, E., and Martí, E. (1999). BDNF and full-length and truncated TrkB expression in Alzheimer disease. Implications in therapeutic strategies. J. Neuropathol. Exp. Neurol. *58*, 729–739.

Golde, T.E. (2003). Alzheimer disease therapy: can the amyloid cascade be halted? J. Clin. Invest. *111*, 11–18.

Hu, X., Das, B., Hou, H., He, W., and Yan, R. (2018). BACE1 deletion in the adult mouse reverses preformed amyloid deposition and improves cognitive functions. J. Exp. Med. *215*, 927–940.

Huang, E.J., and Reichardt, L.F. (2003). Trk receptors: roles in neuronal signal transduction. Annu. Rev. Biochem. *72*, 609–642.

Isshiki, H., Akira, S., Tanabe, O., Nakajima, T., Shimamoto, T., Hirano, T., and Kishimoto, T. (1990). Constitutive and interleukin-1 (IL-1)-inducible factors interact with the IL-1-responsive element in the IL-6 gene. Mol. Cell. Biol. *10*, 2757–2764.

Kaptein, A., Paillard, V., and Saunders, M. (1996). Dominant negative stat3 mutant inhibits interleukin-6-induced Jak-STAT signal transduction. J. Biol. Chem. *271*, 5961–5964.

Kitiyanant, N., Kitiyanant, Y., Svendsen, C.N., and Thangnipon, W. (2012). BDNF-, IGF-1- and GDNF-secreting human neural progenitor cells rescue amyloid β-induced toxicity in cultured rat septal neurons. Neurochem. Res. *37*, 143–152.

Künzi, M.S., and Pitha, P.M. (1996). Role of interferon-stimulated gene ISG-15 in the interferon-omega-mediated inhibition of human immunodeficiency virus replication. J. Interferon Cytokine Res. *16*, 919–927.

Lambert, W.S., Carlson, B.J., Formichella, C.R., Sappington, R.M., Ahlem, C., and Calkins, D.J. (2017). Oral Delivery of a Synthetic Sterol Reduces

Axonopathy and Inflammation in a Rodent Model of Glaucoma. Front. Neurosci. *11*, 45.

Li, M., Makkinje, A., and Damuni, Z. (1996). The myeloid leukemia-associated protein SET is a potent inhibitor of protein phosphatase 2A. J. Biol. Chem. *271*, 11059–11062.

Liu, Z., Jang, S.W., Liu, X., Cheng, D., Peng, J., Yepes, M., Li, X.J., Matthews, S., Watts, C., Asano, M., et al. (2008). Neuroprotective actions of PIKE-L by inhibition of SET proteolytic degradation by asparagine endopeptidase. Mol. Cell *29*, 665–678.

Liu, Y., Liu, A., Li, H., Li, C., and Lin, J. (2011). Celecoxib inhibits interleukin-6/interleukin-6 receptor-induced JAK2/STAT3 phosphorylation in human hepatocellular carcinoma cells. Cancer Prev. Res. (Phila.) *4*, 1296–1305.

Lommatzsch, M., Zingler, D., Schuhbaeck, K., Schloetcke, K., Zingler, C., Schuff-Werner, P., and Virchow, J.C. (2005). The impact of age, weight and gender on BDNF levels in human platelets and plasma. Neurobiol. Aging *26*, 115–123.

Magalini, A., Savoldi, G., Ferrari, F., Garnier, M., Ghezzi, P., Albertini, A., and Di Lorenzo, D. (1995). Role of IL-1 beta and corticosteroids in the regulation of the C/EBP-alpha, beta and delta genes in vivo. Cytokine *7*, 753–758.

Matrone, C., Ciotti, M.T., Mercanti, D., Marolda, R., and Calissano, P. (2008). NGF and BDNF signaling control amyloidogenic route and Abeta production in hippocampal neurons. Proc. Natl. Acad. Sci. USA *105*, 13139–13144.

Minichiello, L. (2009). TrkB signalling pathways in LTP and learning. Nat. Rev. Neurosci. *10*, 850–860.

Murer, M.G., Boissiere, F., Yan, Q., Hunot, S., Villares, J., Faucheux, B., Agid, Y., Hirsch, E., and Raisman-Vozari, R. (1999). An immunohistochemical study of the distribution of brain-derived neurotrophic factor in the adult human brain, with particular reference to Alzheimer's disease. Neuroscience *88*, 1015–1032.

Ni, C.W., Hsieh, H.J., Chao, Y.J., and Wang, D.L. (2004). Interleukin-6-induced JAK2/STAT3 signaling pathway in endothelial cells is suppressed by hemodynamic flow. Am. J. Physiol. Cell Physiol. *287*, C771–C780.

O'Sullivan, L.A., Liongue, C., Lewis, R.S., Stephenson, S.E., and Ward, A.C. (2007). Cytokine receptor signaling through the Jak-Stat-Socs pathway in disease. Mol. Immunol. *44*, 2497–2506.

Peng, S., Garzon, D.J., Marchese, M., Klein, W., Ginsberg, S.D., Francis, B.M., Mount, H.T., Mufson, E.J., Salehi, A., and Fahnestock, M. (2009). Decreased brain-derived neurotrophic factor depends on amyloid aggregation state in transgenic mouse models of Alzheimer's disease. J. Neurosci. *29*, 9321–9329.

Phillips, H.S., Hains, J.M., Armanini, M., Laramee, G.R., Johnson, S.A., and Winslow, J.W. (1991). BDNF mRNA is decreased in the hippocampus of individuals with Alzheimer's disease. Neuron *7*, 695–702.

Poli, V. (1998). The role of C/EBP isoforms in the control of inflammatory and native immunity functions. J. Biol. Chem. *273*, 29279–29282.

Rios, M., Fan, G., Fekete, C., Kelly, J., Bates, B., Kuehn, R., Lechan, R.M., and Jaenisch, R. (2001). Conditional deletion of brain-derived neurotrophic factor in the postnatal brain leads to obesity and hyperactivity. Mol. Endocrinol. *15*, 1748–1757.

Rohe, M., Synowitz, M., Glass, R., Paul, S.M., Nykjaer, A., and Willnow, T.E. (2009). Brain-derived neurotrophic factor reduces amyloidogenic processing through control of SORLA gene expression. J. Neurosci. *29*, 15472–15478.

Selkoe, D.J. (1996). Amyloid beta-protein and the genetics of Alzheimer's disease. J. Biol. Chem. *271*, 18295–18298.

Strohmeyer, R., Shelton, J., Lougheed, C., and Breitkopf, T. (2014). CCAAT-enhancer binding protein-β expression and elevation in Alzheimer's disease and microglial cell cultures. PLoS One *9*, e86617.

Tong, L., Balazs, R., Soiampornkul, R., Thangnipon, W., and Cotman, C.W. (2008). Interleukin-1 beta impairs brain derived neurotrophic factor-induced signal transduction. Neurobiol. Aging *29*, 1380–1393.

Wang, Z.F., Li, Q., Liu, S.B., Mi, W.L., Hu, S., Zhao, J., Tian, Y., Mao-Ying, Q.L., Jiang, J.W., Ma, H.J., et al. (2014). Aspirin-triggered Lipoxin A4 attenuates mechanical allodynia in association with inhibiting spinal JAK2/STAT3 signaling in neuropathic pain in rats. Neuroscience *273*, 65–78.

Wang, Z.-H., Gong, K., Liu, X., Zhang, Z., Sun, X., Wei, Z.Z., Yu, S.P., Manfredsson, F.P., Sandoval, I.M., Johnson, P.F., et al. (2018a). C/EBPβ regulates delta-secretase expression and mediates pathogenesis in mouse models of Alzheimer's disease. Nat. Commun. *9*, 1784.

Wang, Z.-H., Wu, W., Kang, S.S., Liu, X., Wu, Z., Peng, J., Yu, S.P., Manfredsson, F.P., Sandoval, I.M., Liu, X., et al. (2018b). BDNF inhibits neurodegenerative disease-associated asparaginyl endopeptidase activity via phosphorylation by AKT. JCI Insight *3*, 99007.

Waters, M.J., and Brooks, A.J. (2015). JAK2 activation by growth hormone and other cytokines. Biochem. J. *466*, 1–11.

Wedel, A., and Ziegler-Heitbrock, H.W. (1995). The C/EBP family of transcription factors. Immunobiology *193*, 171–185.

Xu, G., Ran, Y., Fromholt, S.E., Fu, C., Yachnis, A.T., Golde, T.E., and Borchelt, D.R. (2015). Murine Aβ over-production produces diffuse and compact Alzheimer-type amyloid deposits. Acta Neuropathol. Commun. *3*, 72.

Yu, H., Kortylewski, M., and Pardoll, D. (2007). Crosstalk between cancer and immune cells: role of STAT3 in the tumour microenvironment. Nat. Rev. Immunol. *7*, 41–51.

Zhang, K., Guo, W., Yang, Y., and Wu, J. (2011). JAK2/STAT3 pathway is involved in the early stage of adipogenesis through regulating C/EBPβ transcription. J. Cell. Biochem. *112*, 488–497.

Zhang, Z., Song, M., Liu, X., Kang, S.S., Kwon, I.S., Duong, D.M., Seyfried, N.T., Hu, W.T., Liu, Z., Wang, J.Z., et al. (2014). Cleavage of tau by asparagine endopeptidase mediates the neurofibrillary pathology in Alzheimer's disease. Nat. Med. *20*, 1254–1262.

Zhang, Z., Song, M., Liu, X., Su Kang, S., Duong, D.M., Seyfried, N.T., Cao, X., Cheng, L., Sun, Y.E., Ping Yu, S., et al. (2015). Delta-secretase cleaves amyloid precursor protein and regulates the pathogenesis in Alzheimer's disease. Nat. Commun. *6*, 8762.

Ziegenhorn, A.A., Schulte-Herbrüggen, O., Danker-Hopfe, H., Malbranc, M., Hartung, H.-D., Anders, D., Lang, U.E., Steinhagen-Thiessen, E., Schaub, R.T., and Hellweg, R. (2007). Serum neurotrophins—a study on the time course and influencing factors in a large old age sample. Neurobiol. Aging *28*, 1436–1445.

# STAR★METHODS

## KEY RESOURCES TABLE

| REAGENT or RESOURCE | SOURCE | IDENTIFIER |
|---|---|---|
| Antibodies | | |
| Anti-BDNF | Novus | NBP2-42215 |
| Rat IL-6 Antibody | R&D | AF-506-SP; RRID:AB_355398 |
| Rat IL-1 beta Antibody | R&D | AF-501-SP; RRID:AB_354508 |
| Rat TNF-alpha Antibody | R&D | AF-510-SP; RRID:AB_354511 |
| Phospho-Stat3 (Tyr705) | CST | 9145; RRID:AB_2491009 |
| Anti-Stat3 | CST | 9139; RRID:AB_331757 |
| Anti-Stat3 (pY705) | BD Biosciences | 612569; RRID:AB_399860 |
| Phospho-Jak2 (Tyr1007/1008) | CST | 3771; RRID:AB_330403 |
| JAK2 Antibody | Bethyl | A302-178A; RRID:AB_1659810 |
| phosho-C/EBPbeta | CST | 3081s; RRID:AB_2244740 |
| phosho-C/EBPβ | CST | 3084s; RRID:AB_2260359 |
| C/EBPβ Antibody (H-7) | Santa Cruz | sc-7962; RRID:AB_626772 |
| Cleaved Caspase-3 (Asp175) Antibody | CST | 9661; RRID:AB_2341188 |
| Anti-Akt pS473 Rabbit mAb | CST | 4060; RRID:AB_2315049 |
| Anti-Akt pT308 | CST | 13038S; RRID:AB_2629447 |
| Anti-AEP 6E3 | Colin Watts Lab | N/A |
| Anti-AEP | R&D Systems | AF2199; RRID:AB_416565 |
| Anti-AEP pT322 | Ye Lab | N/A |
| Anti-APP N585 | Ye Lab | N/A |
| Anti-Tau N368 | Ye Lab | N/A |
| Anti-α-Tubulin | Sigma-Aldrich | T6074; RRID:AB_477582 |
| Anti-beta-actin | Abcam | ab8227; RRID:AB_2305186 |
| Anti-TrkB | R&D | MAB397; RRID:AB_2298820 |
| Anti- TrkB pY706 | Santa Cruz | sc-135645; RRID:AB_2236299 |
| Anti- TrkB pY816 | Ye Lab | N/A |
| Anti-Phospho-Tau Ser202, Thr205 (AT8) | Thermo Fisher | MN1020; RRID:AB_223647 |
| Purified anti-β-Amyloid, 17-24 Antibody | BioLegend | 800712; RRID:AB_2734548 |
| Bacterial and Virus Strains | | |
| AAV-GFP (AAV serotype 2) AAV (titer: 1x10ˆ13 GC/ml) | Vector Biolabs | Cat. No: 7004 |
| AAV-Cre-GFP (AAV serotype 2) AAV (titer: 1x10ˆ13 GC/ml) | Vector Biolabs | Cat. No: 7016 |
| STAT3 Dominant Negative (Y705F) Lentivirus (LV, titer: $8 \times 10^9$ IU/ml) | Kerafast | EH0008 |
| LV- shC/EBPbeta-GFP (titer: $3 \times 10^9$ IU/ml) | viral vector core (VVC) of Emory Universitiy | N/A |
| LV-GFP (titer: $5 \times 10^9$ IU/ml) | viral vector core (VVC) of Emory Universitiy | N/A |
| AAV-AEP C189S | Fredric P. Manfredsson lab | N/A |
| AAV-Control | Fredric P. Manfredsson lab | N/A |
| Biological Samples | | |
| Post-mortem brain samples | Emory Alzheimer's Disease Research Center | N/A |
| BACE1 KO mouse brain samples | Dr. Riqiang Yan's lab | N/A |
| Chemicals, Peptides, and Recombinant Proteins | | |
| rBDNF | Peprotech | Cat# 450-02 |
| DAPI | Sigma-Aldrich | D9542 |
| AEP substrate Z-Ala-Ala-Asn-AMC | Bachem | N/A |

(*Continued on next page*)

*Continued*

| REAGENT or RESOURCE | SOURCE | IDENTIFIER |
|---|---|---|
| β-Secretase Inhibitor | Calbiochem | 171601 |
| DAPT (γ-Secretase Inhibitor) | Calbiochem | 565770 |
| Critical Commercial Assays | | |
| Histostain-SP IHC Kit, mouse | Thermo Fisher | 956543B |
| CytoTox 96® Non-Radioactive Cytotoxicity Assay | Promega | G1780 |
| Rat Beta Amyloid 1-40 ELISA Kit | Lifespan Biosciences | LS-F24096 |
| Rat Beta Amyloid 1-42 ELISA Kit | Lifespan Bioscience | LS-F23254 |
| Amyloid beta 40 ELISA Kit, Mouse | Thermo Fisher | KMB3481 |
| Amyloid beta 42 ELISA Kit, Mouse | Thermo Fisher | KMB3441 |
| IL-1 alpha Mouse ELISA Kit | Thermo Fisher | 88-5019-22 |
| IL-1 alpha Rat ELISA Kit | Thermo Fisher | BMS627TEN |
| IL-6 Mouse ELISA Kit | Thermo Fisher | BMS603HS |
| IL-6 Rat ELISA Kit | Thermo Fisher | BMS625 |
| TNF alpha Mouse ELISA Kit | Thermo Fisher | BMS607-3 |
| TNF alpha Rat ELISA Kit | Thermo Fisher | KRC3011 |
| BDNF ELISA Kit | Abcam | ab212166 |
| Experimental Models: Organisms/Strains | | |
| $BDNF^{f/f}$ mice | Jackson Laboratory | 004339 |
| $TrkB^{f/f}$ mice | Dr. James O McNamara lab | N/A |
| Software and Algorithms | | |
| FV10-ASW | Olympus | N/A |
| ImageJ | https://imagej.nih.gov/ij/ | N/A |
| GraphPad Prism 5 | https://www.graphpad.com/ | N/A |
| GraphPad Prism 6 | https://www.graphpad.com/ | N/A |

## LEAD CONTACT AND MATERIALS AVAILABILITY

Further information and request for resources and reagents should be directed to and will be fulfilled by the Lead Contact, Dr. Keqiang Ye (kye@emory.edu).

## EXPERIMENTAL MODEL AND SUBJECT DETAILS

### Mice

$BDNF^{f/f}$ mice were ordered from the Jackson Laboratory (Stock No: 004339). $TrkB^{f/f}$ mice (gifts from Dr. James O McNamara at Duke University, USA) were in a C57BL/6J background. $BDNF^{f/f}$ and $TrkB^{f/f}$ mice were aged 13.5 months at time of brain injection. The following animal experiments were conducted on these mice at the age of 16 months. Animal care and handling was performed according to the NIH animal care guidelines and Emory Medical School guidelines. The protocol was reviewed and approved by the Emory Institutional Animal Care and Use Committee. All procedures performed in studies involving animals were in accordance with the ethical standards of the Emory Institutional Animal Care and Use Committee. Both male and female mice were used. Animals were equally divided into groups for each sex.

### Primary cultured neurons

Primary cortical neurons were cultured as previously described (Zhang et al., 2014). All rats were purchased from the Jackson Laboratory. The protocol was reviewed and approved by the Emory Institutional Animal Care and Use Committee. Cells were incubated at 37 °C in a humidified atmosphere of 5% $CO_2$. Primary cortical neurons were isolated from embryonic E18 Sprague–Dawley rats. Briefly, tissues were dissected, dissociated and incubated with 5 ml of D-Hanks containing 0.25% trypsin for 5 min, centrifuged at 1000 × g for 5 min after addition of 4 ml of the neuronal plating medium containing DMEM/F12 with 10% fetal bovine serum. Then the cells were resuspended, about $5 \times 10^5$ cells were plated onto each well of 12-well plates for western blotting, and $1 \times 10^5$ cells were plated onto each glass coverslip for cell imaging. The neurons were then put into a humidified incubator with 5% $CO_2$ at 37 °C. The medium was changed to neurobasal medium supplemented with 2% B27 (maintenance medium) after 2–4 h.

### Human tissue samples

Post-mortem brain samples were dissected from frozen brains of AD and aged-match non-demented controls from the Emory Alzheimer's Disease Research Center. The study was approved by the Biospecimen Committee. AD was diagnosed according to the criteria of the Consortium to Establish a Registry for AD and the National Institute on Aging. Diagnoses were confirmed by the presence of amyloid plaques and neurofibrillary tangles in formalin-fixed tissue. Informed consent was obtained from the subjects. The information of the human case materials:

Control 1: Age at Death:70, ApoE3/3, male, Braak Score: I;
Control 2: Age at Death:75, ApoE3/3, female, Braak Score: I;
Control 3: Age at Death:69, ApoE3/3, male, Braak Score: I I;
Control 4: Age at Death:74, ApoE3/3, female, Braak Score: I;
Control 5: Age at Death:79, ApoE3/3, male, Braak Score: I I;
AD 1: Age at Death:71, ApoE3/4, male, Braak Score: VI;
AD 2: Age at Death:77, ApoE3/4, female, Braak Score: I I I;
AD 3: Age at Death:69, ApoE3/4, male, Braak Score: VI;
AD 4: Age at Death:71, ApoE4/4, female, Braak Score: V;
AD 5: Age at Death:77, ApoE4/4, male, Braak Score: VI;
AD 6: Age at Death:72, ApoE4/4, male, Braak Score: VI;

## METHOD DETAILS

### Transfection and infection of the cells

LV- shC/EBPbeta-GFP (titer: $3 \times 10^9$ IU/ml) and LV-GFP (titer: $5 \times 10^9$ IU/ml): Coding sequence of shC/EBPbeta was inserted into pFCGW-N1 lentiviral vectors (CMV promoter). The virus was packaged by viral vector core (VVC) of Emory Universitiy. (titer: $1x10^{13}$ GC/ml) and (titer: $1x10^{13}$ GC/ml): Viral genomes were generated by inserting the coding sequence behind the neuronal synapsin promoter. Viral vectors were packaged in to AAV2/9 via triple transfection and purified using an iodixanol gradient followed by column chromatography. Titers were determined using a dot-blot assay. 3 μL of virus was added to 1 mL culture medium and applied to primary neurons.

### BDNF deprivation

All experimental treatments were performed on 8- to 9-day-old cultures. Neurons were treated with BDNF (50 ng/ml) for 48 h 3–5 days after plating, after which the medium was rinsed and cultures were washed three times with neurotrophin-free medium and then incubated for another 48h in the same medium also containing anti-BDNF antibody. Treatment with anti-BDNF (Mab BDNF, 30 μg/ml; Novus, NBP2-42215) was carried out for 48 h. Then the cell lysates were prepared for immunoblotting, or coated slides were fixed for IF staining.

### Cytokines blocked by neutralizing antibodies

To establish whether the release of IL-1β, IL-6, and TNFα to the culture medium was mechanistically connected to the BDNF deprivation-mediated STAT3 activation and C/EBPβ expression, we performed experiments in which the three cytokines were blocked by neutralizing antibodies added to the cultured medium. We cocultured neurons for 24 h in the presence of neutralizing antibodies against IL-1β, IL-6, and TNFα(1 μg/ml; R&D), and then exposed the cultures to anti-BDNF or anti-IgG for another 48 h.

### AEP activity assay

Tissue homogenates or cell lysates (10 μg) were incubated in 200 μL reaction buffer (20 mM citric acid, 60 mM $Na_2HPO_4$, 1 mM EDTA, 0.1% CHAPS and 1 mM DTT, pH 5.5) containing 20 μM AEP substrate Z-Ala-Ala-Asn-AMC (Bachem). AMC released by substrate cleavage was quantified by measuring at 460 nm in a fluorescence plate reader at 37°C in kinetic mode.

### Immunoprecipitation and western blot analysis

Cells were washed with ice-cold PBS and lysed in Co-immunoprecipitation (Co-IP) buffer (50 mM Tris–HCl [pH 7.5], 150 mM NaCl, 1% Nonidet P-40, 5 mM EDTA, 5 mM EGTA, 15 mM $MgCl_2$, 60 mM β-glycerophosphate, 0.1 mM sodium orthovanadate, 0.1 mM NaF, 0.1 mM benzamide, 10 mg/ml aprotinin, 10 mg/ml leupeptin and 1 mM PMSF) at 4 °C for 2 h with rotation. Immunoprecipitated proteins were separated by SDS-PAGE and then transferred to a nitrocellulose membrane. The membrane was blocked with Tris-buffered saline (TBS) containing 5% nonfat milk and 0.1% Tween 20 (TBST) at RT for 1 h, followed by the incubation with primary antibody at 4 °C overnight, and with the secondary antibody at RT for 1 h. After washing with TBST, the membrane was developed using the enhanced chemiluminescent (ECL) detection system.

### Immunohistochemistry

Immunohistochemistry was performed by using the peroxidase protocol. Briefly, tissue sections were deparaffinized in xylene, hydrated through descending ethanol concentrations, and endogenous peroxidase activity was eliminated by incubation in 3%

hydrogen peroxide in methanol for 5 min. After antigen-retrieval in boiling sodium citrate buffer (10 mM), the sections were incubated with primary antibodies for overnight at 4 °C. The signal was developed using Histostain-SP kit (Invitrogen). For the double immunofluorescence staining, the sections were incubated overnight at 4 °C with a mixture of antibodies. After being washed with TBS, the sections were incubated with a mixture of Alexa Fluor 488-/594- and CY5-coupled secondary antibodies (Invitrogen) for detection. Images were acquired through Confocal (Olympus FV1000).

### Electron Microscopy

After deep anesthesia, mice were perfused transcardially with 2% glutaraldehyde and 3% paraformaldehyde in PBS. Hippocampal slices were post-fixed in cold 1% $OsO_4$ for 1 h. Samples were prepared and examined using standard procedures. Ultrathin sections (90 nm) were stained with uranyl acetate and lead acetate and viewed at 100 kV in a JEOL 200CX electron microscope. Synapses were identified by the presence of synaptic vesicles and postsynaptic densities.

### Stereotaxic injection in mouse hippocampus

Animals received bilateral injections of virus. Animals were anesthetized with intraperitoneal injections of 100/10 mg/kg ketamine/xylazine and given 0.1 mg/kg buprenorphine subcutaneously for pain management. Depth of anesthesia was assessed via toe-pinch. The mice had their heads shaved and restrained in a stereotaxic frame with mouse adaptor (Stoeltling, Wood Dale, IL). Sterile Bausch & Lomb erythromycin ophthalmic ointment (0.5%) was applied to the eyes to keep them from drying out, and their heads cleaned with 70% ethanol. A small incision was made in the skin down the midline of the cranium, exposing the skull landmarks lambda and Bregma. Hydrogen peroxide was used to clean the top of the skull. Target injection coordinates were mapped from Bregma, and a small hole drilled through the skull directly above target sites with a bone drill. Hippocampal coordinates were anteroposterior (AP) $-2.1$ mm, mediolateral (ML) $\pm$ 1.5 mm, dorsoventral (DV) 1.8 mm. (1 $\mu$L of AAV or/and 3 $\mu$L of LV)Injection rate was 0.2 $\mu$L per minute, and the needle was left in place for 10-min post-injection. After injections, the incision was sutured and Neosporin applied. Animals recovered on heating pads until awake and monitored 1, 2, 7, and 10 days post-surgery. All surgeries were performed with IACUC approval. Mice were assigned into gender- and age- matched treatment groups using a randomized block design.

### Golgi staining

Mouse brains were fixed in 10% formalin for 24 h, and then immersed in 3% potassium bichromate for 3 days in the dark. The solution was changed each day. Then the brains were transferred into 2% silver nitrate solution and incubated for 24 h in the dark. Vibratome sections were cut at 60 mm, air-dried for 10 min, dehydrated through 95% and 100% ethanol, cleared in xylene and coverslipped.

### Electrophysiology

Mice were anaesthetized with isoflurane, decapitated, and their brains dropped in ice-cold artificial cerebrospinal fluid (a-CSF) containing 124 mM NaCl, 3 mM KCl, 1.25 mM $NaH_2PO_4$, 6.0 mM $MgCl_2$, 26 mM $NaHCO_3$, 2.0 mM $CaCl_2$, and 10 mM glucose. Hippocampi were dissected and cut into 400-mm thick transverse slices with a vibra- tome. After incubation at room temperature (23-24 °C) in aCSF for 60-90 min, slices were placed in a recording chamber (RC-22C, Warner Instruments) on the stage of an up-right microscope (Olympus CX-31) and perfused at a rate of 3 mL per min with a-CSF (containing 1 mM $MgCl_2$) at 23-24 °C. A 0.1 MU tungsten monopolar electrode was used to stimulate the Schaffer collaterals. The field excitatory post-synaptic potentials (fEPSPs) were recorded in CA1 stratum radiatum by a glass microelectrode filled with a-CSF with resistance of 3-4 MU. The stimulation output (Master-8; AMPI, Jerusalem) was controlled by the trigger function of an EPC9 amplifier (HEKA Elektronik, Lambrecht, Germany). fEPSPs were recorded under current-clamp mode. Data were filtered at 3 kHz and digitized at sampling rates of 20 kHz using Pulse software (HEKA Elektronik). The stimulus intensity (0.1 ms duration, 10–30 mA) was set to evoke 40% of the maximum f-EPSP and the test pulse was applied at a rate of 0.033 Hz. LTP of fEPSPs was induced by 3 theta-burst-stimulation (TBS), it is 4 pulses at 100 Hz, repeated 3 times with a 200-ms interval). The magnitudes of LTP are expressed as the mean percentage of baseline fEPSP initial slope.

### Morris Water maze

Mice were trained in a round, water-filled tub (52 inches diameter) in an environment rich with extra maze cues as described previously (Zhang et al., 2014). The water maze was surrounded by extramaze visual cues that remained in the same position for the duration of training and filled to cover the platform by 1 cm at 22 °C. Water was made opaque with nontoxic, white tempera paint. The escape platform was a circular, nonskid surface (area 127 $cm^2$) placed in the NW quadrant of the maze. Each subject was given 4 trials/day for 5 consecutive days with a 15 min intertrial interval. The maximum trial length was 60 s, and if subjects did not reach the platform in the allotted time, they were manually guided to it. Following the 5 d of task acquisition, a probe trial was presented, during which time the platform was removed and the percentage of time spent in the quadrant that previously contained the escape platform during task acquisition was measured over 60 s. All trials were analyzed for latency and swim speed by means of MazeScan (Clever Sys).

### Contextual fear conditioning

The ability to form and retain an association between an aversive experience and environmental cues was tested with a standard fear conditioning paradigm that occurs over a period of 3 d. Mice were placed in the fear conditioning apparatus (7'' W, 7'' D 3 12'' H, Coulbourn) composed of Plexiglass with a metal shock grid floor and allowed to explore the enclosure for 3 min. Following this habituation period, 3 conditioned stimulus (CS)-unconditioned stimulus (US) pairings were presented with a 1 min intertrial interval. The CS was composed of a 20 s, 85-dB tone and US was composed of 2 s of a 0.5-mA footshock, which was co-terminate with each CS presentation. One minute following the last CS-US presentation, mice were returned to their home cage. On day 2, the mice were presented with a context test, during which subjects were placed in the same chamber used during conditioning on day 1 and the amount of freezing was recorded via a camera and the software provided by Coulbourn. No shocks were given during the context test. On day 3, a tone test was presented, during which time subjects were exposed to the CS in a novel compartment. Initially, animals were allowed to explore the novel context for 2 min. Then the 85-db tone was presented for 6 min and the amount of freezing behavior was recorded.

## QUANTIFICATION AND STATISTICAL ANALYSIS

All data are expressed as mean ± s.e.m. from three or more independent experiments and analyzed using GraphPad Prism statistical software (GraphPad Software). All of the statistical details of experiments can be found in the figure legends for each experiment, including the statistical tests used, number of mice in animal experiments (represented as n, unless otherwise stated), number of wells in cell culture experiments (represented as n, unless otherwise stated), definition of center (mean). Sample size was determined by Power and Precision (Biostat). The level of significance between two groups was assessed with unpaired t test with Welch's correction. For more than two groups, one-way ANOVA and Bonferroni's multiple comparison test was applied. The two-way ANOVA and Bonferroni's post hoc test compared the differences between groups that have been split on two independent factors. A value of $p < 0.05$ was considered to be statistically significant.

**Cell Reports, Volume *28***

## Supplemental Information

# Deficiency in BDNF/TrkB Neurotrophic Activity Stimulates δ-Secretase by Upregulating C/EBPβ in Alzheimer's Disease

**Zhi-Hao Wang, Jie Xiang, Xia Liu, Shan Ping Yu, Fredric P. Manfredsson, Ivette M. Sandoval, Shengxi Wu, Jian-Zhi Wang, and Keqiang Ye**

## Supplementary Figure 1

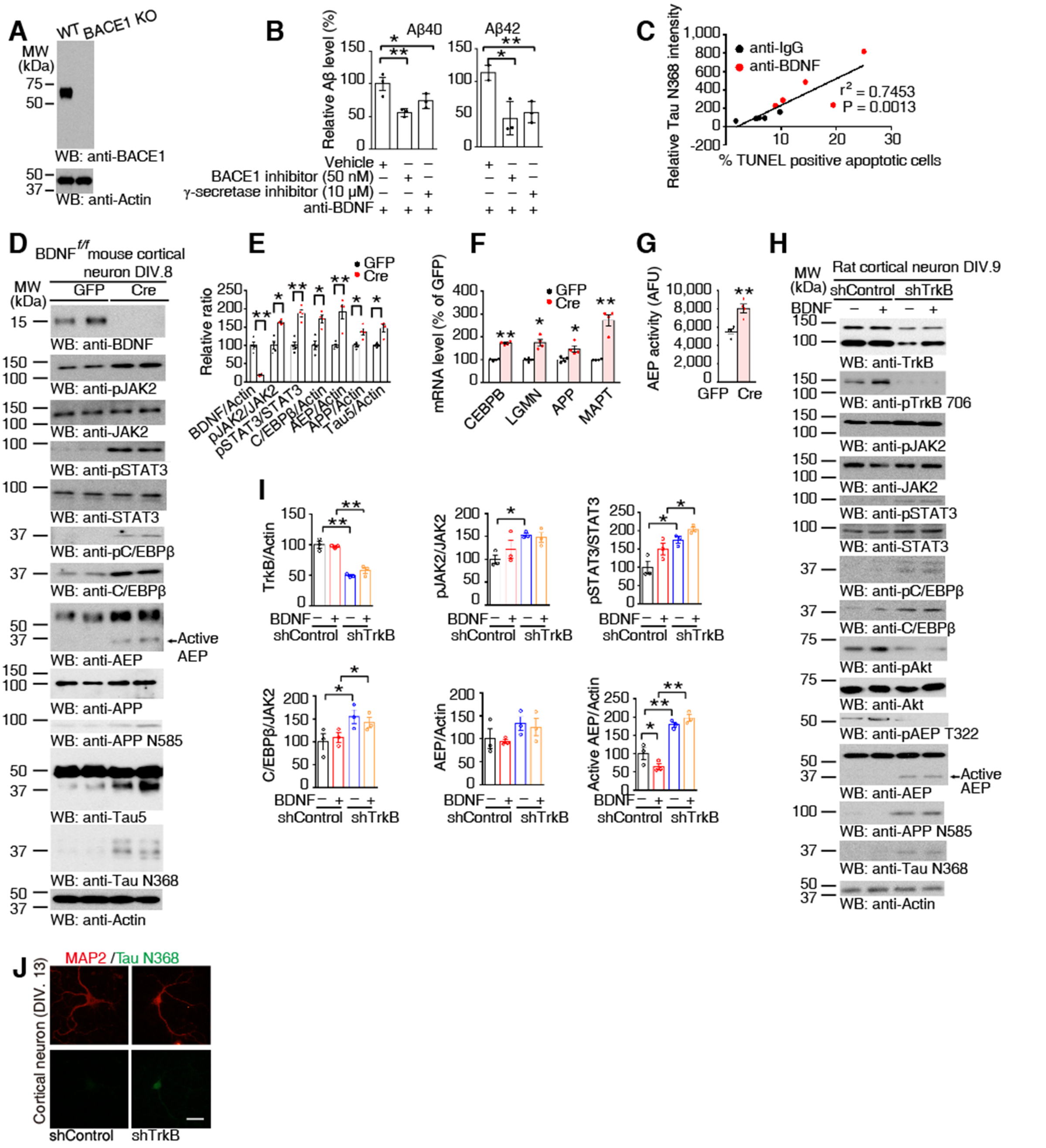


**Fig. S1 BDNF/TrkB depletion activates JAK2/STAT3, increasing C/EBP expression. Related to Figure 1.**

**A-B** Validation of anti-BACE1 antibody and Aβ ELISA kits (mean ± s.e.m., n = 3, *P < 0.05, **P < 0.01, one-way ANOVA and Bonferroni's multiple comparison test and Bonferroni's multiple comparison test). **C** TUNEL and immunofluorescence double-labeling assay showing BDNF deprivation-induced neurotoxicity

directly correlates with Tau N368 level. The Spearman correlation coefficient $r^2$ and $p$ value are shown. **D** BDNF depletion activates JAK2/STAT3 and increases the expressions of C/EBPβ and δ-secretase. Immunoblotting was conducted from $BDNF^{f/f}$ mice neurons transduced with AAV-Cre or AAV-GFP. Western blot data are representative of three independent experiments. **E** Quantification of western blotting results in Fig. S1D (mean ± s.e.m., n = 4, *P < 0.05, **P < 0.01, unpaired t-test with Welch's correction). **F** Quantitative RT-PCR analysis of CEBPB, LGMN, APP and MAPT mRNA levels in $BDNF^{f/f}$ neurons transduced with AAV-Cre or AAV-GFP. Data represent mean ± s.e.m. (n = 4, *P < 0.05, **P < 0.01, unpaired t-test with Welch's correction). **G** BDNF deprivation stimulates δ-secretase enzymatic activities. Data represent mean ± s.e.m. (n = 4, **P < 0.01, unpaired t-test with Welch's correction). **H** TrkB depletion activates JAK2/STAT3 and increases the expression of C/EBPβ and δ-secretase. Immunoblotting was conducted from neurons transduced with vectors expressing shRNA against TrkB or control shRNAs. BDNF (100 ng/ml) was supplied for 20 minutes before cell harvest. Western blot data are representative of three independent experiments. **I** Quantification of western blotting results (mean ± s.e.m., n = 3, **P < 0.01, unpaired t-test with Welch's correction). **J** IF staining showing an increase of Tau cleavage induced by TrkB depletion. Scale bar, 30 μm.

## Supplementary Figure 2

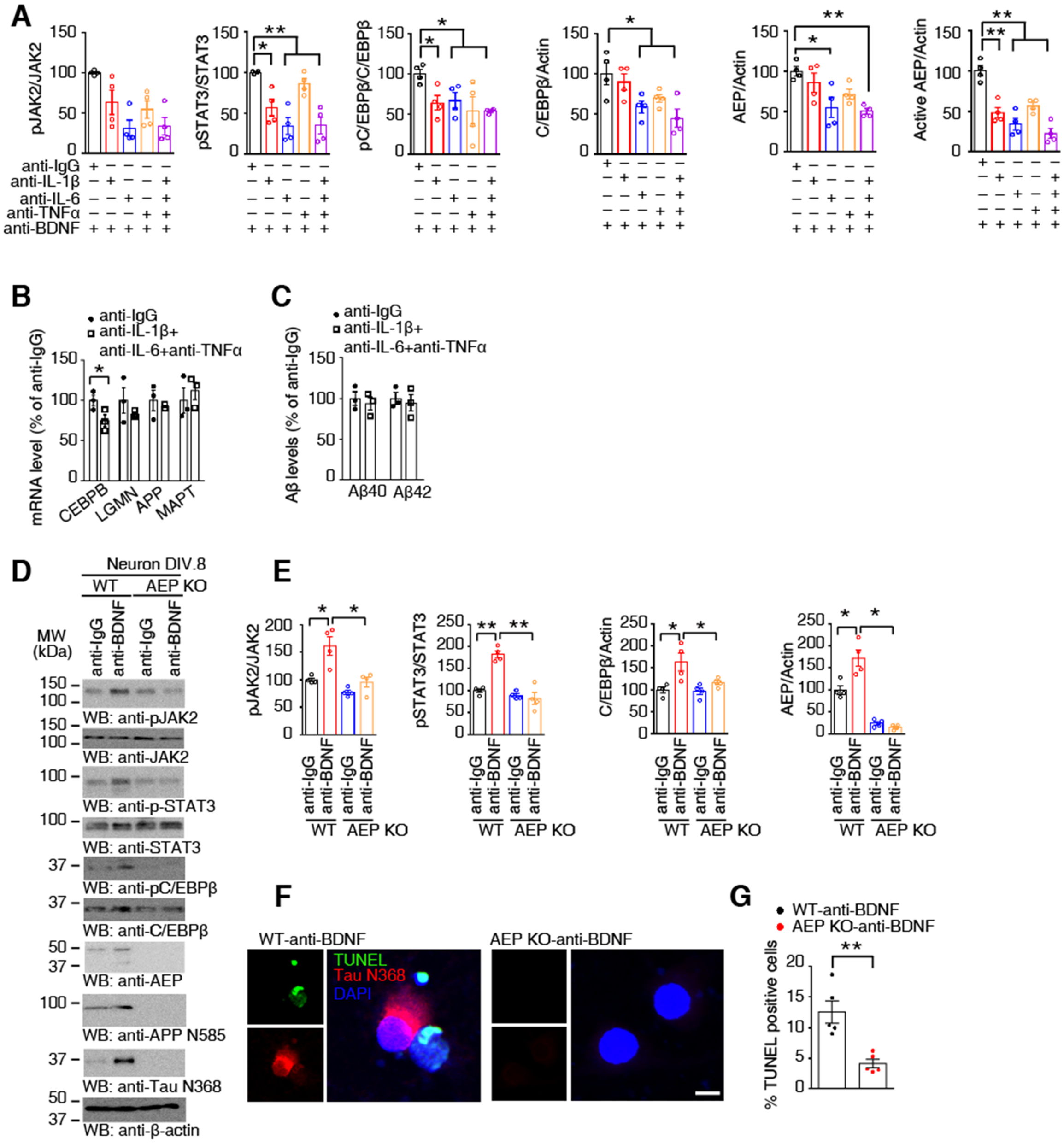


**Fig. S2 Cytokine neutralization or AEP knockout inhibits JAK2/STAT3 and decreases the expression of C/EBPβ and delta-secretase. Related to Figure 2 and 3.**

**A** Quantification of western blotting results from BDNF-deprived rat cortical neurons pretreated with various cytokine antibodies (n = 4, *P < 0.05, **P < 0.01, one-way ANOVA and Bonferroni's multiple comparison test). **B-C** Effects of anti-inflammatory cytokine treatment on CEBPB, LGMN, APP, MAPT, and Aβ levels without anti-BDNF treatment (n = 3, *P < 0.05, one-way ANOVA and Bonferroni's multiple comparison test). **D** AEP

knockout prevents the cleavage of Tau and APP induced by BDNF deprivation. **E** Quantification of western blotting results in Fig. S3D (n = 4, *P < 0.05, **P < 0.01, one-way ANOVA and Bonferroni's multiple comparison test). **F-G** TUNEL and IF double-staining showing BDNF deprivation-induced neurotoxicity is repressed by AEP knockout. Scale bar, 10 μm. Quantification of TUNEL positive cells represents mean ± s.e.m. (n = 5 slides, **P < 0.01, unpaired t-test with Welch's correction).

## Supplementary Figure 3

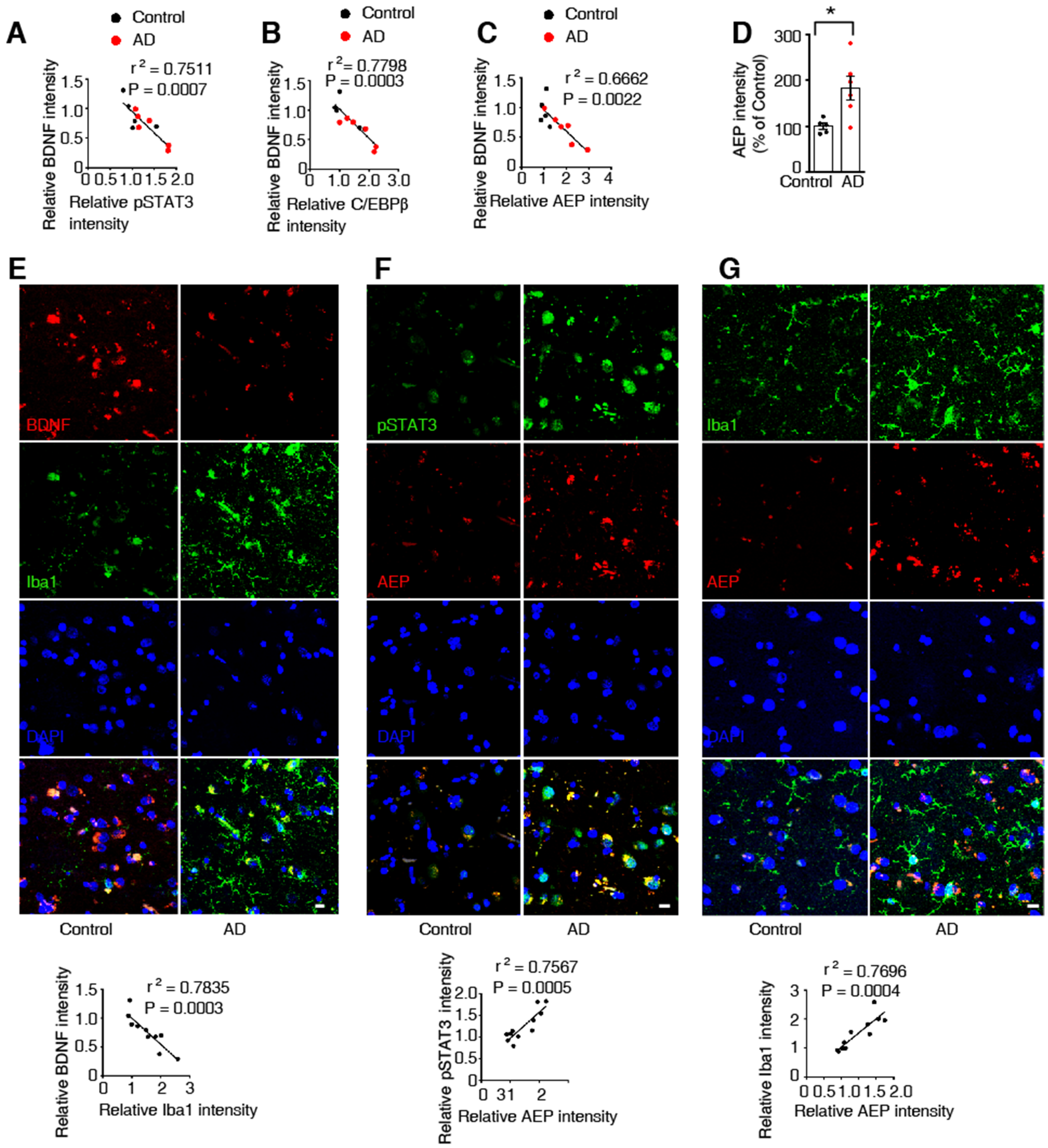


**Fig. S3 Direct correlation between BDNF deficiency, neuroinflammation, and β-secretase upregulation in human AD brains. Related to Figure 4.**

**A-D** Quantitative analysis of the fluorescent intensity signals revealing direct correlation between BDNF and each of p-STAT3, C/EBPβ and δ-secretase. The Spearman correlation coefficient $r^2$ and $p$ value are shown. Quantification of AEP intensity represents mean ± s.e.m. (n=5 cases for control; n=6 cases for AD; *P < 0.05,

**P < 0.01, unpaired t-test with Welch's correction). **E-G** IF staining of BDNF/Iba1/pSTAT3/AEP in human hippocampus samples. IF data are representatives of 5 or 6 independent cases. Scale bar, 10 μm. Quantitative analysis of the fluorescent intensity signals revealing direct correlation between BDNF and Iba1, p-STAT3 and AEP, Iba1 and AEP. The Spearman correlation coefficient $r^2$ and $p$ value are shown.

## Supplementary Figure 4

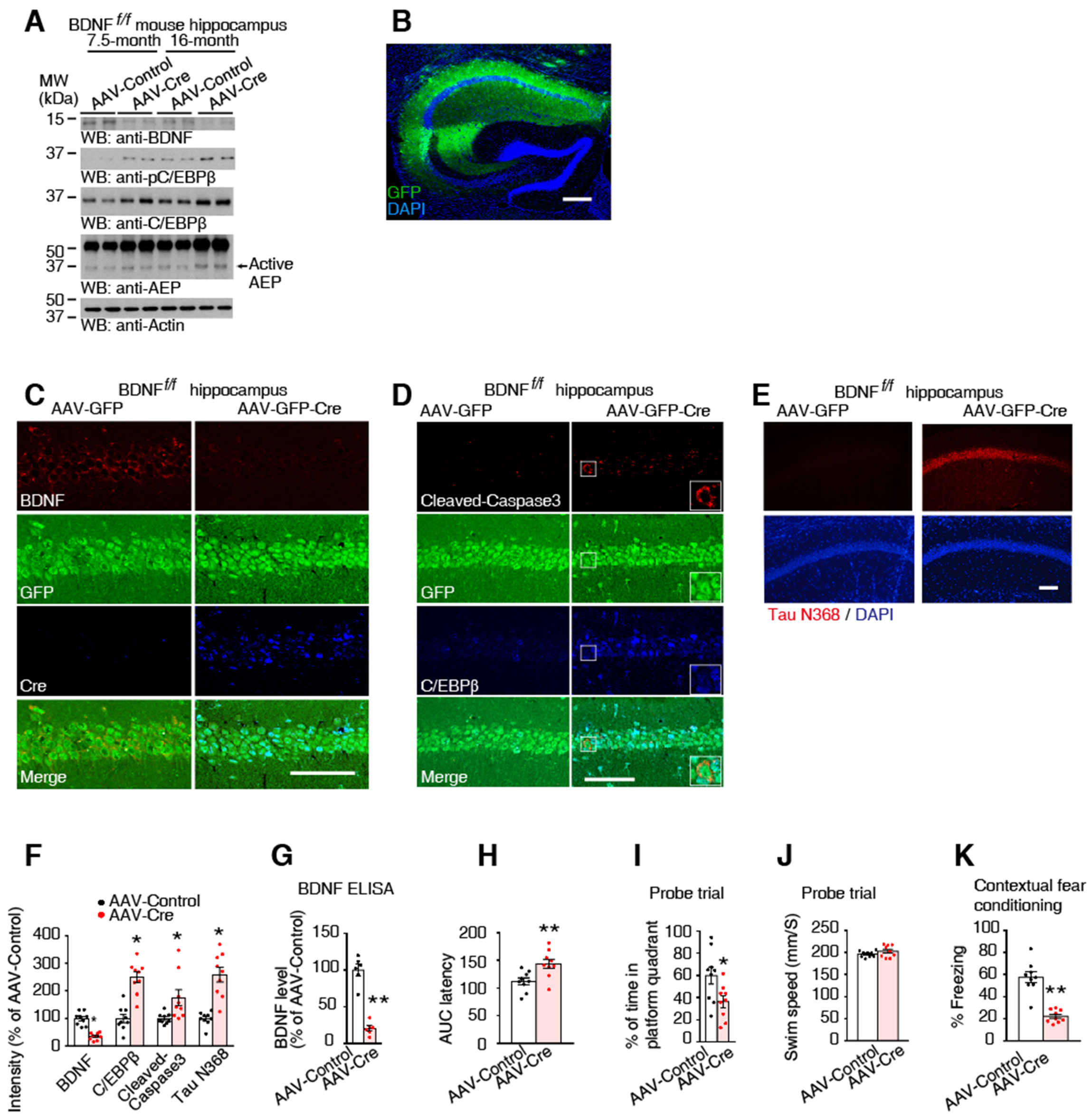


**Fig. S4 BDNF knockout in hippocampus triggers murine Aβ and tau alterations. Related to Figure 5.**

**A** BDNF knockout induced C/EBPβ/AEP activation in mice. **B** AAV-mediated GFP expression in BDNF$^{f/f}$ mice hippocampus. Scale bar, 200 μm. **C-E** IF staining of BDNF and Cre, cleaved-Caspase3 and C/EBPβ and Tau N368 in BDNF$^{f/f}$ mice hippocampal CA1 transduced with AAV-GFP or AAV-Cre. Scale bar, 100 μm. **F** Quantification of fluorescent intensity represent mean ± s.e.m. (n = 9 sections from 3 mice, *P < 0.05, unpaired t-test with Welch's correction). **G** Relative level of BDNF by ELISA (mean ± s.e.m.; n = 5 mice per group;

**P<0.01, unpaired t-test with Welch's correction)**. H** Integrated latency (AUC) for mice (mean ± s.e.m.; n = 9 mice per group; **P<0.01, unpaired t-test with Welch's correction)**. I** Morris Water Maze analysis. BDNF knockout impaired memory (mean ± s.e.m.; n = 8-9 mice per group; *P< 0.05, unpaired t-test with Welch's correction). **J** The swim speed of mice in MWM test remained comparable (mean ± s.e.m.; n = 9 mice per group, unpaired t-test with Welch's correction). **K** Fear conditioning test. Contextual fear conditioning was reduced in BDNF knockout mice (mean ± s.e.m.; n = 9 mice per group; **P<0.01, unpaired t-test with Welch's correction)**.**

## Supplementary Figure 5

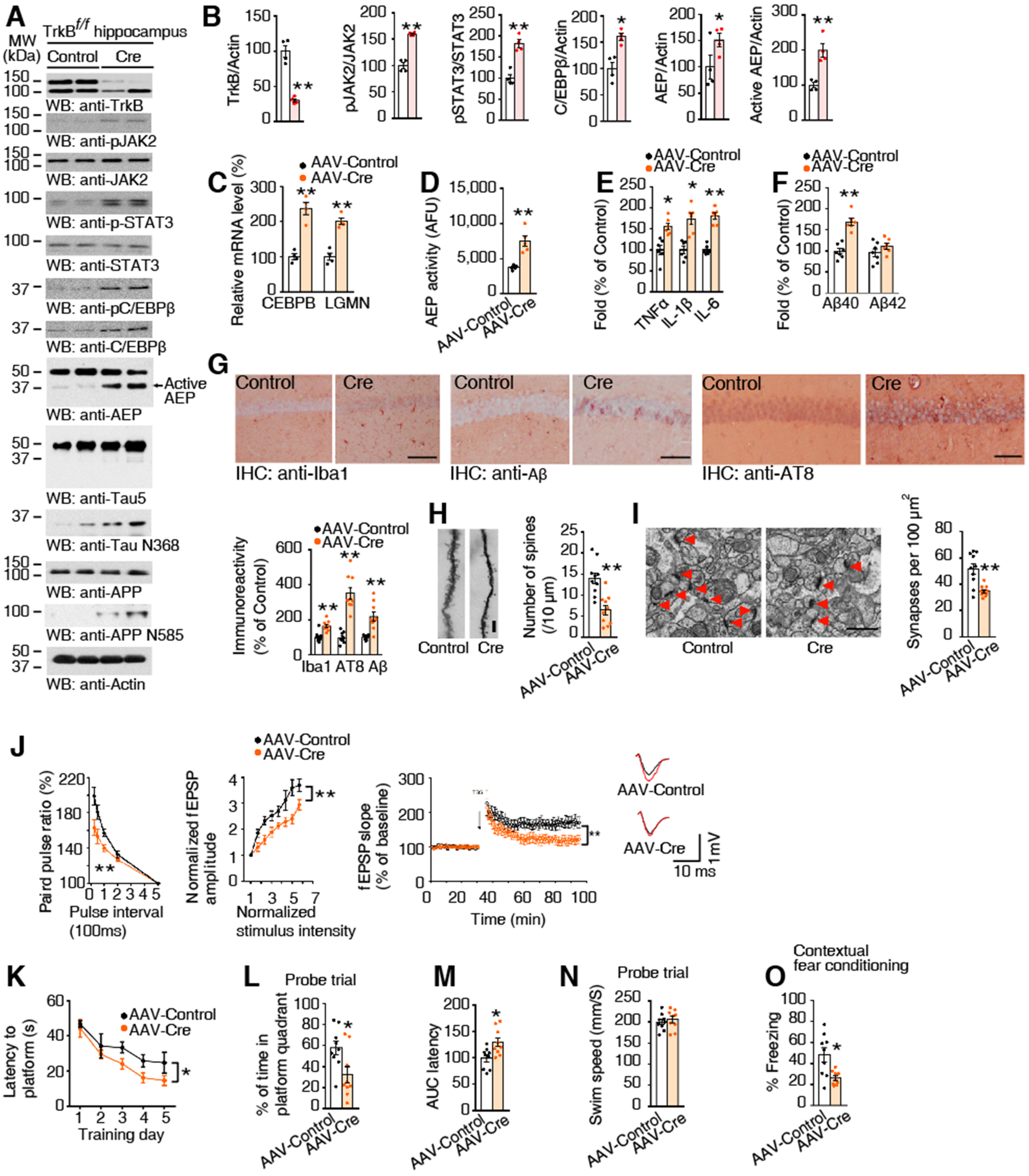


**Fig. S5 Knockout of TrkB activates the JAK2/STAT3 pathway and induces murine Aβ and tau alterations. Related to Figure 5.**

**A** TrkB knockout activates JAK2/STAT3 and increases the expression of C/EBPβ and δ-secretase. Immunoblotting was conducted from 16-month-old TrkB$^{f/f}$ mice hippocampus injected with AAV-Cre or AAV-GFP. Western blot data are representative of three independent experiments. **B** Quantification of western

blotting results in Fig. S5A (n = 4 mice, *P < 0.05, **P < 0.01, unpaired t-test with Welch's correction). **C** Quantitative RT-PCR analysis of CEBPB and LGMN mRNA levels in hippocampus with TrkB knockout. Data represent mean ± s.e.m. (n = 4, **P < 0.01, unpaired t-test with Welch's correction).**D** TrkB knockout increases δ-secretase enzymatic activities. Data represent mean ± s.e.m. (n = 5, *P < 0.05, unpaired t-test with Welch's correction). **E** TrkB knockout stimulates inflammatory cytokine production. Data represent mean ± s.e.m. (n = 6, *P < 0.05, **P < 0.01, unpaired t-test with Welch's correction). **F** TrkB knockout increases Aβ production. Quantification of Aβ40 and Aβ42 levels by ELISA represents mean ± s.e.m. (n = 6-7 mice, *P < 0.05, **P < 0.01, unpaired t-test with Welch's correction). **G** IHC of anti-Iba1, anti-Aβ and anti-AT8 showing that TrkB knockout promotes mouse Aβ and tau alterations. Scale bar, 50 μm. Data shown as mean ± s.e.m. of 10 sections from three mice (*P < 0.05, unpaired t-test with Welch's correction). **H** TrkB knockout decreases the dendritic spine density. Golgi staining was conducted on brain sections from apical dendritic layer of the CA1 region. Scale bar, 5 μm. Data represent mean ± s.e.m. of 10 sections from 3 mice in each group. (*P < 0.05, unpaired t-test with Welch's correction). **I** EM analysis demonstrating that the synapses are decreased in the TrkB knockout hippocampus. Scale bar, 1 μm. Data represent mean ± s.e.m. of 10 sections from 3 mice in each group. (*P < 0.05, unpaired t-test with Welch's correction). **J** Electrophysiology analysis. TrkB knockout leaded to LTP defects. The ratio of paired pulses in different groups (mean ± s.e.m.; n = 6 in each group; **P < 0.01, two-way ANOVA) (left). Input-output curve (middle) represent mean ± s.e.m. of 6 mice per group (**P < 0.01, two-way ANOVA). LTP of fEPSPs (mean ± s.e.m.; n = 6 in each group; **P < 0.01, two-way ANOVA) (right). Shown traces are representative fEPSPs recorded before (black) and 60 minutes after (red) TBS. **K-M** Morris Water Maze analysis. TrkB knockout impaired the learning and memory (mean ± s.e.m.; n = 9 mice per group; *P< 0.05, two-way ANOVA for **K** and unpaired t-test with Welch's correction for **L** and **M**). **N** The swim speed of mice in MWM test remained comparable (mean ± s.e.m.; n = 9 mice per group, unpaired t-test with Welch's

correction). **O** Fear condition test. Contextual fear conditioning was reduced in TrkB knockout mice (mean ± s.e.m.; n = 9 mice per group; *P<0.05, unpaired t-test with Welch's correction).

## Supplementary Figure 6

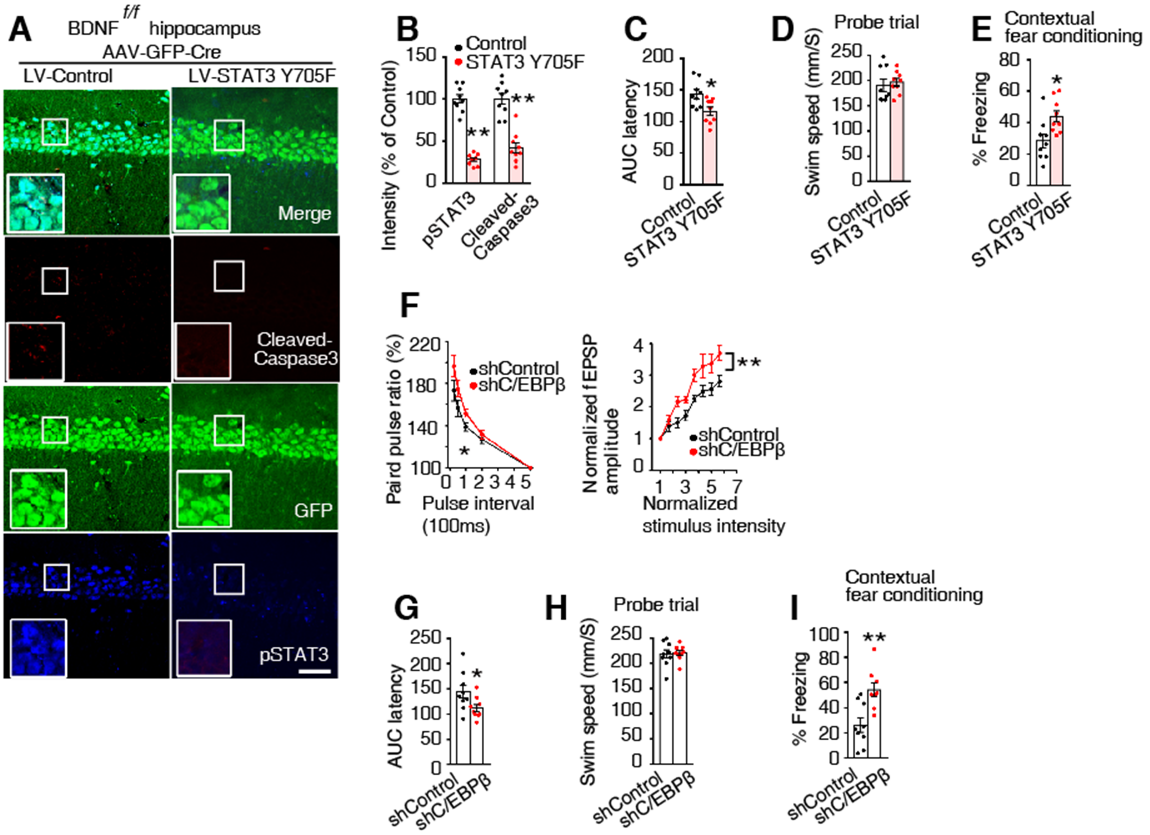


**Fig. S6 STAT3 Y705F mutant expression rescues BDNF depletion-mediated neuronal apoptosis and cognitive deficits. Related to Figure 6 and 7.**

**A** IF staining of cleaved-Caspase 3 and pSTAT3 in BDNF flox/flox mice hippocampal CA1 injected with AAV-Cre + LV-Control or AAV-Cre + LV-STAT3 Y705F. Scale bar, 50 μm. **B** Quantification of fluorescent intensity represents mean ± s.e.m. (n = 9 sections from 3 mice, *P < 0.05, unpaired t-test with Welch's correction). **C** Integrated latency (AUC) for mice (mean ± s.e.m.; n = 9 mice per group; *P < 0.05, unpaired t-test with Welch's correction)**. D** The swim speed of mice in MWM test remained comparable (mean ± s.e.m.; n = 9 mice per group, unpaired t-test with Welch's correction)**. E** Fear condition test. Contextual fear conditioning was reduced in BDNF knockout mice (mean ± s.e.m.; n = 9 mice per group; *P<0.05, unpaired t-test with Welch's correction)**. F** Electrophysiology analysis. The ratio of paired pulses (lift) in different groups (mean ± s.e.m.; n = 6 in each group; *P < 0.05, two-way ANOVA and Bonferroni's post hoc test). Input-output

curves (right) represent mean ± s.e.m. of 6 mice per group (*$P < 0.05$, two-way ANOVA and Bonferroni's post hoc test). **G** Integrated latency (AUC) for mice (mean ± s.e.m.; n = 9 mice per group; *$P < 0.05$, unpaired t-test with Welch's correction)**. H** The swim speed of mice in MWM test remained comparable (mean ± s.e.m.; n = 9 mice per group, unpaired t-test with Welch's correction)**. I** Fear conditioning test. Contextual fear conditioning was reduced in BDNF knockout mice (mean ± s.e.m.; n = 9 mice per group; **$P < 0.01$, unpaired t-test with Welch's correction)**.**

# Supplementary Figure 7

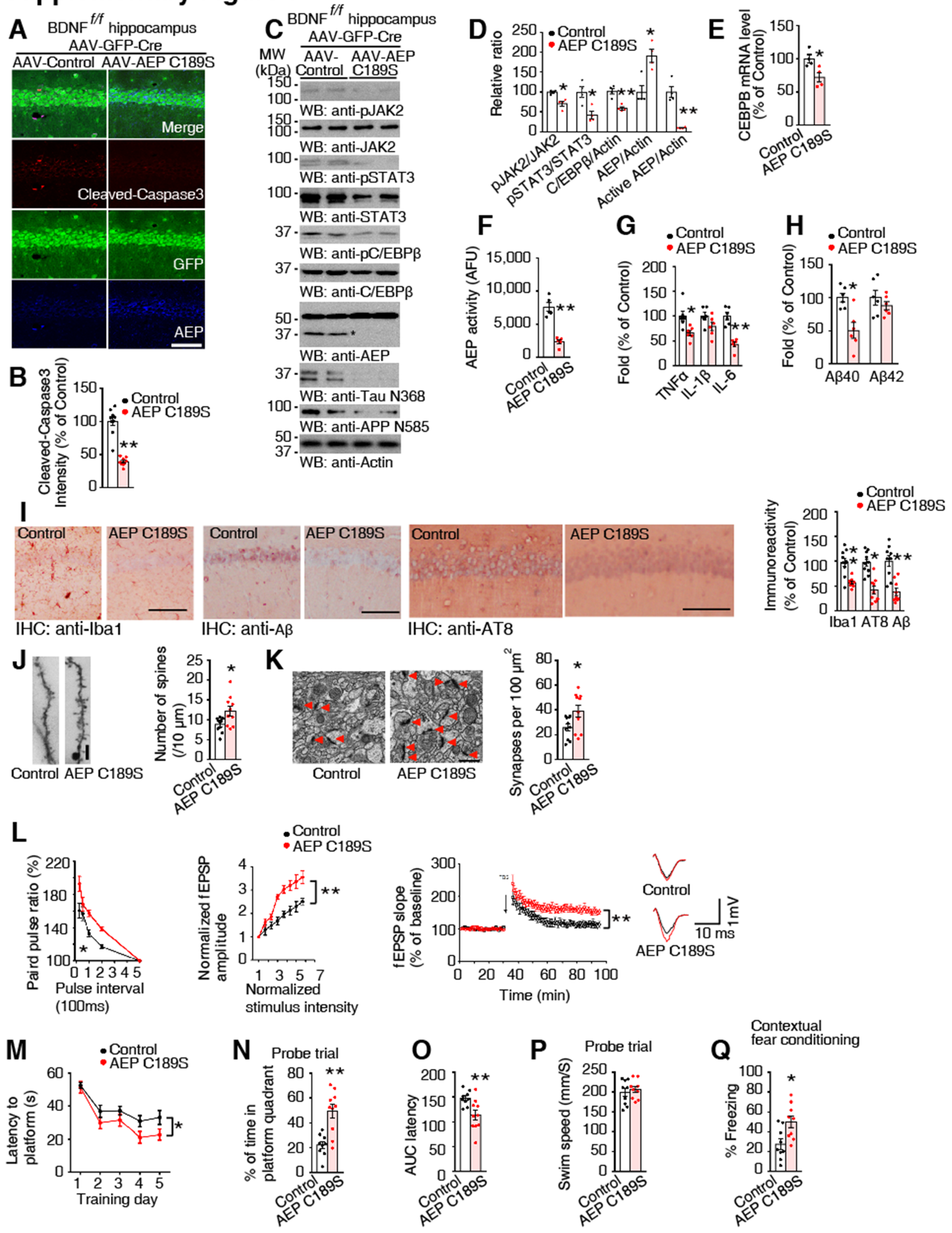


**Fig. S7 Inhibition of delta-secretase represses BDNF depletion-induced neuronal apoptosis and murine Aβ and tau alterations. Related to Figure 7.**

**A-B** IF staining of cleaved-Caspase 3 and AEP in BDNF flox/flox mice hippocampal CA1 injected with AAV-Cre + AAV-Control or AAV-Cre +AAV-AEP C189S. Scale bar, 50 μm. Quantification of fluorescent intensity represents mean ± s.e.m. (n = 9 sections from 3 mice, *P < 0.05, unpaired t-test with Welch's correction). **C** AAV-AEP C189S expression prevents δ-secretase activation in the BDNF-depleted hippocampus. Immunoblotting was conducted from 16-month-old BDNF flox/flox mice hippocampus co-injected with AAV-Cre and AAV-Control or AAV-Cre and AAV-AEP C189S. Western blot data are representative of three independent experiments. *Active AEP. **D** Quantification of western blotting results (n = 4 mice, *P < 0.05, **P < 0.01, unpaired t-test with Welch's correction). **E** Quantitative RT-PCR analysis of CEBPB mRNA levels in $BDNF^{f/f}$ mice hippocampus. Data represent mean ± s.e.m. of four mice (**P < 0.01, unpaired t-test with Welch's correction). **F** BDNF knockout-induced activation of δ-secretase is lessened by AEP C189S expression. Data represent mean ± s.e.m. of five mice (*P < 0.05, unpaired t-test with Welch's correction). **G** Relative cytokine levels measured by ELISA of hippocampus lysates. Data represent mean ± s.e.m. of six samples per group (*P < 0.05, **P < 0.01, unpaired t-test with Welch's correction). **H** Inhibition of δ-secretase represses Aβ production induced by BDNF knockout. Quantification of Aβ40 and Aβ42 levels by ELISA represents mean ± s.e.m. (n = 6 mice,*P < 0.05, **P < 0.01, unpaired t-test with Welch's correction). **I** Immunohistochemistry (IHC) of anti-Iba1, anti-Aβ and anti-AT8 in hippocampal CA1. Scale bar, 50 μm. Data shown as mean ± s.e.m. of 6 sections from three mice (*P < 0.05, unpaired t-test with Welch's correction).**J** AEP C189S increases the dendritic spine density. Golgi staining was conducted on brain sections from apical dendritic layer of the CA1 region. Scale bar, 5 μm. Data on the right represent mean ± s.e.m. of 10 sections from 3 mice in each group. (*P < 0.05, unpaired t-test with Welch's correction). **K** EM analysis. Scale bar, 1 μm. Data represent mean ± s.e.m. of 10 sections from 3 mice in each group. (*P < 0.05, unpaired t-test with Welch's correction). **L** Electrophysiology analysis. AEP C189S rescued LTP defects. The ratio of paired pulses in different groups

(mean ± s.e.m.; n = 6 in each group; *P < 0.05, two-way ANOVA and Bonferroni's post hoc test) (left). Input-output curve (middle) represent mean ± s.e.m. of 6 mice per group (**P < 0.01, two-way ANOVA and Bonferroni's post hoc test). LTP of fEPSPs (mean ± s.e.m.; n = 6 mice in each group; **P < 0.01, two-way ANOVA and Bonferroni's post hoc test). Shown traces are representative fEPSPs recorded before (black) and 60 minutes after (red) TBS. **M-Q** Morris Water Maze and Fear conditioning. AEP C189S ameliorated the learning and memory impairment (mean ± s.e.m.; n = 9 mice per group; *P< 0.05, **P< 0.01, two-way ANOVA and Bonferroni's post hoc test for **n** and unpaired t-test with Welch's correction for **o-r**).
